# Metatranscriptomic analysis of diverse microbial communities reveals core metabolic pathways and microbiome-specific functionality

**DOI:** 10.1186/s40168-015-0146-x

**Published:** 2016-01-12

**Authors:** Yue Jiang, Xuejian Xiong, Jayne Danska, John Parkinson

**Affiliations:** Program in Molecular Structure and Function, The Hospital for Sick Children, Peter Gilgan Center for Research and Learning, 686 Bay Street, Toronto, ON M5G 0A4 Canada; Department of Immunology, University of Toronto, Toronto, ON Canada; Program in Genetics and Genomic Biology, The Hospital for Sick Children, Toronto, ON Canada; Department of Medical Biophysics, University of Toronto, Toronto, ON Canada; Departments of Biochemistry, Computer Science and Molecular Genetics, University of Toronto, Toronto, ON Canada; Centre for the Analysis of Genome Evolution, University of Toronto, Toronto, ON Canada

**Keywords:** Metatranscriptomics, Bioinformatics, Systems biology, Next generation sequencing, RNA sequencing, Microbiome

## Abstract

**Background:**

Metatranscriptomics is emerging as a powerful technology for the functional characterization of complex microbial communities (microbiomes). Use of unbiased RNA-sequencing can reveal both the taxonomic composition and active biochemical functions of a complex microbial community. However, the lack of established reference genomes, computational tools and pipelines make analysis and interpretation of these datasets challenging. Systematic studies that compare data across microbiomes are needed to demonstrate the ability of such pipelines to deliver biologically meaningful insights on microbiome function.

**Results:**

Here, we apply a standardized analytical pipeline to perform a comparative analysis of metatranscriptomic data from diverse microbial communities derived from mouse large intestine, cow rumen, kimchi culture, deep-sea thermal vent and permafrost. Sequence similarity searches allowed annotation of 19 to 76 % of putative messenger RNA (mRNA) reads, with the highest frequency in the kimchi dataset due to its relatively low complexity and availability of closely related reference genomes. Metatranscriptomic datasets exhibited distinct taxonomic and functional signatures. From a metabolic perspective, we identified a common core of enzymes involved in amino acid, energy and nucleotide metabolism and also identified microbiome-specific pathways such as phosphonate metabolism (deep sea) and glycan degradation pathways (cow rumen). Integrating taxonomic and functional annotations within a novel visualization framework revealed the contribution of different taxa to metabolic pathways, allowing the identification of taxa that contribute unique functions.

**Conclusions:**

The application of a single, standard pipeline confirms that the rich taxonomic and functional diversity observed across microbiomes is not simply an artefact of different analysis pipelines but instead reflects distinct environmental influences. At the same time, our findings show how microbiome complexity and availability of reference genomes can impact comprehensive annotation of metatranscriptomes. Consequently, beyond the application of standardized pipelines, additional caution must be taken when interpreting their output and performing downstream, microbiome-specific, analyses. The pipeline used in these analyses along with a tutorial has been made freely available for download from our project website: http://www.compsysbio.org/microbiome.

**Electronic supplementary material:**

The online version of this article (doi:10.1186/s40168-015-0146-x) contains supplementary material, which is available to authorized users.

## Background

Next generation sequencing technologies have revolutionized the study of complex microbial communities (microbiomes). In the context of human health, composition of the intestinal microbiome has been linked with type I diabetes, inflammatory bowel disease and obesity [1–3]. Many such studies focus on microbial community composition using marker genes such as 16S ribosomal RNA (rRNA) to survey the relative abundance of individual taxa [4–6]. Since multiple combinations of microbial taxa can confer similar metabolic outputs, efforts have begun to define microbiome function through untargeted RNA sequencing (metatranscriptomics) [7–10]. For example, metatranscriptomic analyses have recently revealed the expression of specialized fermentation genes in the production of kimchi [9] and methylamine degradation pathways in the rumen of the cow [8].

Illumina sequencing platforms have emerged as leading technologies for metatranscriptomic analysis. In addition to the volume of sequence reads generated, annotation of these complex data is further challenged due to the relatively short sequence lengths [11]. Overcoming these issues requires identification and removal of sequence reads from library adaptors, ribosomal RNA or other sequencing artefacts, transcript assembly, assignment of reads to known functions and taxa and tools that allow the intuitive visualization of the results. To date, metatranscriptomic studies have tended to use a variety of customized scripts and tools to perform filtering, assembly and sequence similarity searches. For example, a kimchi transcriptome dataset used BLASTN sequence similarity searches to filter rRNA reads, the SEED database [12] for functional annotation and BWA software [13] to map reads to reference genomes of six representative lactic acid bacterial strains previously associated with the kimchi microbial community [9]. Results were visualized with heatmaps showing the relative expression of genes involved in carbohydrate metabolism. A bovine metatranscriptome study focused on the rumen [8], assembled sequence reads using the SHE-RA software [14] performed taxonomic assignments with BLASTX searches against the Genbank RefSeq protein database [15, 16] and functional annotations using the SEED database. Thus, in the absence of analyses being performed using a single standardized software solution, it has been difficult to compare the results of different studies and identify microbiome-specific taxonomic and functional signatures.

A key question is how availability of high quality reference genomes and the complexity of a microbial community impact sequence annotation and inference of biological insight. The broad functional classification schemes in resources such as KEGG, COG and SEED [17–19] provide limited molecular level characterization. Moreover, the field needs to develop statistical approaches that capture significant gene expression differences across metatranscriptomes. To address these limitations, we developed and applied a single standardized pipeline analysis to compare five microbiomes from diverse habitats: deep-sea, permafrost, cow’s rumen, kimchi and mouse cecal content. Our results demonstrate how integration of taxonomic and functional data within a novel visualization framework can provide insight into the taxonomic contributions to biochemical pathways.

## Results and discussion

### Annotation of metatranscriptomic datasets reflect depth of available reference genomes

**Fig. 1:**
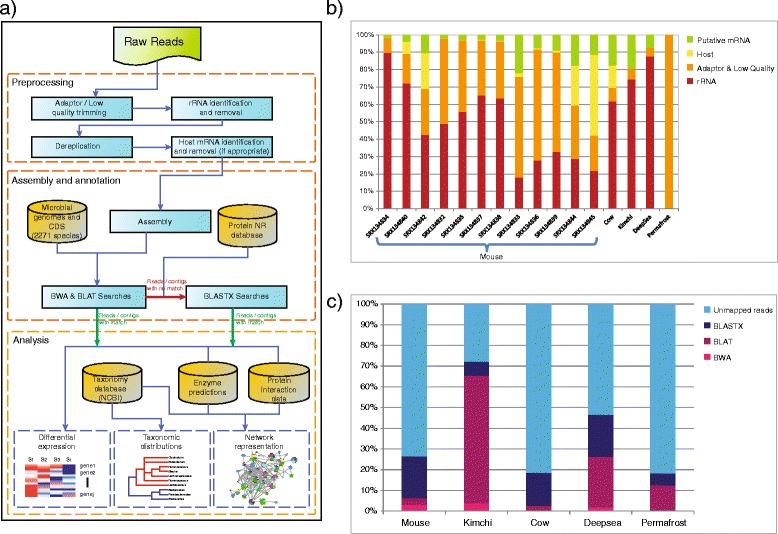
Workflow and Read Processing. **a** Workflow of the pipeline for processing, annotation and analyses of metatranscriptome (RNA-seq). **b** Composition of sequence reads for twelve mouse metatranscriptome datasets and four additional microbiomes (see Methods). **c** Distribution of reads annotated through three complementary sequence similarity search tools: (1) BWA and (2) BLAT searches against a database of 2271 microbial genomes and (3) BLASTX searches against the protein non-redundant database. The mouse dataset represents a summary of all 12 datasets analysed in this study

We applied a systematic pipeline to process sequence data from five metatranscriptomic studies: (1) 30 million 76 bp paired end reads from 12 mouse large intestine samples [7]; (2) 35 million 101 bp single end reads from a sample of kimchi, obtained on the 29th day of fermentation [9]; (3) 14 million 100 bp paired end reads from a sample obtained from a bovine rumen [8]; (4) 103 million 100 bp paired end reads from a deep-sea sample [10]; and (5) 131 million 150 bp paired end reads from a sample obtained from permafrost (Fig. 1a). All datasets were generated with Illumina sequencing platforms. After the removal of rRNA/tRNA, low quality, adaptor contaminants and host sequences, from 0.01 % (permafrost) to 19.1 % (kimchi) messenger RNA (mRNA) reads were predicted (Fig. 1b and Additional file 1). The permafrost sample was composed of 99.9 % low quality and adaptor reads, likely reflecting the low biomass of this sample. In addition, mouse intestinal content samples (prepared with Invitrogen mirVana kit) displayed higher proportions of reads of host origin (23 and 47 %) relative to other samples (0.3–21 %), reflecting the abundance of epithelial cell shedding in this compartment. In the absence of a complete set of reference genomes to which reads could be effectively mapped, read assembly can help improve annotation. For each dataset, putative mRNA reads were assembled using the Trinity RNA-Seq assembly algorithm [20] which we previously identified as an optimal short read assembler for metatranscriptomic data, in terms of improving annotation as well as minimizing the incidence of misassemblies [11]. The deep-sea and kimchi datasets possessed the highest proportion of reads assembled (‘contigs’; 62 and 72 %, respectively). The kimchi dataset featured a contig N50 length of 368 bp, likely reflecting the limited diversity of this microbiome.

Assembled contigs and unassembled reads, representing putative mRNA sequences, were then parsed through a hierarchical annotation pipeline, with unannotated reads passing to the next annotation step. This analysis included (1) mapping of sequences to a reference set of 4443 prokaryotic genomes using the BWA algorithm that relies on near perfect sequence matches (defined here as no more than two base pair mismatches—see Methods) [13]; (2) sequence similarity searches against the same set of reference genomes using a less stringent BLAT algorithm [21]; and (3) sequence similarity searches against the protein non-redundant database [22] using BLASTX [23]. Of the five datasets, the cow rumen samples produced the lowest frequency (19 %) and the kimchi dataset featured the highest frequency (72 %) of mapped reads (Fig. 1c). This latter result is a consequence of 51 % of putative mRNA reads that were mapped to two reference genomes, *Lactobacillus sakei* and *Weissella koreensis*. The high proportion of BLAT mappings compared to BWA results suggests genetic variation from the reference strains. BLAT-based mapping identified 24 % of the deep sea, and 12 % of the permafrost datasets, but mapping of the mouse gut and cow rumen samples reads performed better with the least stringent BLASTX algorithm (Fig. 1c). These findings highlight the lack of representative reference genomes for these microbiomes, such that many sequence reads map to homologs from distant relatives of the actual species present in the samples.

These results are broadly consistent with the original reports of these datasets but also highlight important differences produced by the selected analytical pipelines. For example, the cow rumen study [8], which relied on BLASTX sequence similarity searches with a score cut-off less than e^-5^, reported ~400,000 reads of putative mRNA origin compared to 452,708 reported here. However, we do note some significant discrepancies. The original study of the kimchi microbiome [9] applied the BWA algorithm to map 3.9 million reads to six reference strains; here, using the BWA/BLAT/BLASTX pipeline, we mapped 4.8 million reads to bacterial mRNA transcripts. For the deep-sea microbiome, the original analysis applied a combination of the Velvet and Oases assembly algorithms to construct 78,000 contigs with an average contig size of 243 bp [10]. Subsequent sequence similarity searches using the BWA algorithm identified ~81,000 predicted genes, of which only 18,500 were protein coding. In the current study, we identified 643,000 contigs with an N50 of 110 bp with the Trinity assembly. Further, we identified 243,000 unique transcripts by inclusion of 3.0 million reads not assigned to a contig. These differences reflect the often arbitrary choice of parameters and algorithms, usually in the absence of rigorous benchmarking, that can impact coverage and accuracy, and highlight the need for standardized pipelines.

### Pathway enrichment analysis identifies tissue specific gene expression in the mouse gut microbiome

In previous studies of the cow rumen, deep-sea and kimchi microbiomes, gene expression was assessed by direct comparisons of raw or normalized read counts [8–10]. In the absence of standardized statistical models to identify significant changes in gene expression from metatranscriptomic datasets, we evaluated three methods previously employed to detect changes in gene expression: DEseq2 [24], EdgeR [25] and ANOVA-like differential expression analysis (ALDEx2—[26]). We compared microbial expression patterns between three cecal wall-associated (cecal wall) and four cecal lumen flush derived (cecal flush) microbiomes from four NOD strain mice of identical age and sex which had been prepared with the same RNA extraction protocol. Of the 20,160 non-mouse transcripts identified in these samples (11,231 and 11,015 for cecal wall and cecal flush, respectively), 2087 were shared between sample types. Only five transcripts displayed significant differences in expression between the two types of microbiome samples (Additional file 2). This reflects the large variation observed across animals and tissue samples as defined by a biological coefficient of variation (BCV) of 1.11, where the BCV is a measure of how the (unknown) true abundance of the gene varies between replicate RNA samples (see Methods).

While the above approaches are useful for identifying individual genes displaying differential expression across samples, additional insights can be gained by considering collections of functionally related genes (e.g. complexes and pathways). We therefore applied a pathway enrichment approach that, due to the limited number of genes identified above, relied on fold change in expression [27, 28], to examine expression of metabolic pathways. In this analysis, 551 genes displayed ≥fivefold difference in expression between the two types of samples, with a greater frequency of genes up-regulated in the cecal wall compared to the cecal flush datasets (Additional file 3). We identified 199 genes that could be mapped to 72 unique Enzyme Commission (EC) identifiers. Pathway enrichment analysis revealed 17 metabolic pathways to be significantly associated with these genes (hypergeometric test, *p* value <0.05; Table 1). Pathways demonstrating significant differential expression include six involved in carbohydrate metabolism (e.g. the citrate cycle, pyruvate metabolism and glycolysis/gluconeogenesis), four involved in amino acid metabolism and three involved in energy metabolism. Previous studies have shown that changes in the expression of carbohydrate-associated enzymes in the mouse intestinal microbiome were linked to microbial community composition [29, 30].

**Table 1:** Pathways enriched in transcripts displaying large (>fivefold) differences in relative expression between mouse cecal wall and cecal flush samples

		Fold change in expression						Differentially expressed genes	Matched ECs/total ECs in pathway
		Genes up-regulated in cecal wall			Genes up-regulated in cecal flush				
Pathway	*p* value	5–10	10–20	>20	5–10	10–20	>20		
Glycolysis/gluconeogenesis	9.35E-07	14	5	3	5	4	1	32	11/45
Methane metabolism	6.44E-05	10	5	3	4	5	2	29	11/68
Carbon fixation in photosynthetic organisms	1.00E-04	8	1	2	4	0	0	15	5/25
One carbon pool by folate	2.88E-04	4	3	0	1	2	0	10	6/24
Starch and sucrose metabolism	4.59E-04	5	4	2	3	3	0	17	10/71
Alanine, aspartate and glutamate metabolism	1.43E-03	9	1	0	0	1	0	11	7/43
Citrate cycle (TCA cycle)	1.52E-03	4	0	2	2	1	0	9	5/22
Pyruvate metabolism	3.08E-03	7	0	2	1	1	1	12	8/62
Amino sugar and nucleotide sugar metabolism	6.63E-03	5	3	1	1	3	2	15	9/85
Oxidative phosphorylation	1.10E-02	2	3	1	0	3	0	9	3/12
Purine metabolism	3.08E-03	10	0	0	2	0	2	14	9/100
Propanoate metabolism	3.37E-02	3	0	0	0	1	1	5	5/45
Valine, leucine and isoleucine biosynthesis	3.40E-02	1	1	0	0	1	2	5	3/18
Aminoacyl-tRNA biosynthesis	3.85E-02	2	0	0	1	0	3	6	4/32
Histidine metabolism	4.25E-02	1	3	0	1	0	0	5	4/33
Drug metabolism—other enzymes	4.49E-02	3	0	0	0	0	0	3	3/20
Other glycan degradation	4.88E-02	1	1	0	0	0	0	2	2/9

### Short read data reveals microbiome-specific taxonomic signatures

**Fig. 2:**
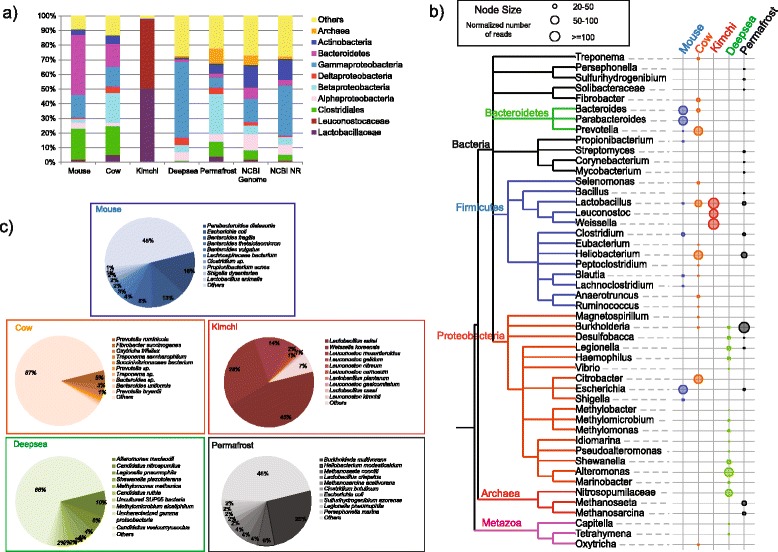
Taxonomic composition of five metatranscriptomic datasets. **a** Abundance of 10 major bacterial phyla and sub-phyla across the five samples. Also shown are the observed frequency of assignments in the 2271 microbial genomes used in the BWA and BLAT searches as well as the protein non-redundant database. **b** Phylogenetic representation of major genera (represented by at least 100 reads) associated with the five datasets. Node size represents the relative abundance of reads mapped to the corresponding genus in each sample. For each dataset, reads were normalized by the average read count associated with each sample (see Methods). **c** Top ten most abundant species associated with each dataset (by number of reads; minimum 100 transcripts)

In addition to deriving functional insights (see following sections), we were interested in the ability of metatranscriptomic datasets, associated with relatively short reads, to inform on the taxonomic composition of a habitat. Based on mappings of reads of putative mRNA origin to known genes, we explored the taxonomic assignments of reads at three different taxonomic levels (Fig. 2). Previous comparisons across the mouse datasets revealed relatively minor taxonomic variations between samples at least at the class/phylum level [7, 11]. Here, we identified distinct taxonomic profiles for each microbiome. At the level of phylum, reads from all five samples could be largely defined into four major groups: *Firmicutes*, *Proteobacteria, Bacteroidetes* and *Actinobacteria* (Fig. 2a). However, while the cow rumen and mouse intestinal samples had significant representation from all four taxa, the kimchi sample was largely restricted to the Firmicute families Leuconostocaceae and Lactobacillaceae, while the deep-sea and permafrost samples lacked significant representation of Bacteriodetes, the former also lacking significant representation of Actinobacteria. Indeed, consistent with a previous study based on 16S rRNA reads [10, 31], we found that the majority (51 %) of reads of putative mRNA origin from the deep-sea sample could be classified as Gammaproteobacteria. Interestingly, we also found reads mapping to non-bacterial genes. For example, for the deep-sea dataset, we identified reads mapping to Archaea (0.8 % of reads of putative mRNA origin), fungi (0.6 %) and protozoa (1.5 %).

Next, we examined the contribution of distinct genera to each microbiome (Fig. 2b). Within these ‘abundant’ genera, the deep-sea dataset displayed the largest number of unique taxa (13) while the kimchi dataset displayed the fewest (2; *Leuconostoc* and *Weissellla*). Indeed, the kimchi dataset appears dominated by three main taxa. On the other hand, *Lactobacillus* was well represented across four of the five datasets; although present in the deep-sea dataset, it does not comprise one of the defined, abundant genera in this dataset. We note that *Lactobacillus* is one of the twelve most abundant genera in our reference datasets (45 genomes) and assignment of a large proportion of reads to this genus may simply reflect that bias, potentially acting as a surrogate taxon for species not represented within our reference datasets. In any event, despite such biases, our pipeline reveals each habitat to possess a unique taxonomic signature with the presence of specific abundant taxa adapted to individual environmental conditions. For example, *Weissella* is a genus of lactic acid bacteria, first identified in kimchi at 2002, that are regarded as one of the three main genera that are strongly associated with fermentation of kimchi based on both transcriptome or 16S rRNA study [9, 32, 33]. This analysis also shows the value in using a higher level of taxonomic resolution. For example, from Fig. 2a, both the cow rumen and mouse samples reveal the presence of reads from Bacteroidetes; however, deeper analysis reveals such reads to be dominated by *Prevotella* in the cow rumen sample compared to *Bacteroides* and *Parabacteroides* in the mouse samples.

Finally, we examined the performance of the hierarchical annotation pipeline to assign reads to discrete species for each sample (Fig. 2c). To reduce the influence of species with matches involving only a limited number of genes, only species represented by 100 or more transcripts were included in these analyses with the exception of the permafrost sample, the latter due to the low number of putative mRNA reads. The kimchi sample was associated with the simplest community, with 10 species accounting for ~93 % of total reads of putative mRNA origin. These assignments were remarkably consistent with a previous report [9], with similar abundances for five of the top six most represented taxa. Emphasizing the findings at the genus level, there was no overlap in the ten most abundant species in the mouse and cow datasets despite the phylum/sub-phylum similarities (Fig. 1a). The mouse microbiome samples were obtained from germ-free animals colonized with altered Schaedler flora (ASF) which were defined, before the advent of high through-put sequence analysis, to contain eight known species [34, 35]: *Lactobacillus acidophilus, Lactobacillus murinis, Parabacteroides distasonis, Mucispirillum schaedleri*, three members of Clostridium cluster XIV and a poorly characterized Firmicute species. Of these previously defined species, only *P. diastonis* appears significantly represented in our samples. However, previous studies have suggested that *Lactobacillus animalis*, identified within the samples, is identical to *L. murinis* [34], while reads assigned to the poorly classified ‘*Clostridium* sp.’ may represent the species associated with Clostridium cluster XIV. The additional species presented in Fig. 2c likely represent close relatives to the remaining unaccounted ASF taxa. Conversely and again consistent with a previous study, amongst the top ten most abundant species represented in the cow dataset were those that have previously been associated with the rumen [36] including bacteria that degrade cellulose and other carbohydrates (*Prevotella* spp*.* and *Fibrobacter* spp.) and those that utilize fatty acids (*Succinivibrionaceae* spp*.* and *Treponema* spp*.*) as well as the protozoan *Oxytricha trifallax*, a relative of *Oxytricha granulifera*, previously reported to occupy the rumen [37]. Similarly, the deep-sea dataset was represented by species previously associated with the marine environment [10, 31] including *Alteromonas macleodi*, the ammonia oxidizing archaeon—*Candidatus nitrospumilis*, methanotrophs (*Methylomonas methanica* and *Methylomicrobium alcaliphilum*) and the sulphur oxidizing SUP05 [38]. Across samples, we note a varying proportion (from 7 to 87 % for kimchi and cow rumen datasets, respectively) of reads mapping to ‘Others’. These include reads from species with few transcripts and likely false positives, as well as reads associated with a more diverse community. For example, we note that in the deep-sea dataset, 504 species were represented by 100 or more transcripts, with species represented by 10 or fewer transcripts contributing only 3.9 % of the reads, suggesting a highly diverse microbiome. On the other hand, only 67 species were represented by 100 or more transcripts in the cow rumen dataset, with 45 % of the reads contributed by species with 10 or fewer transcripts, suggesting a higher number of false positive assignments. Beyond resorting to more complex phylogenetic mapping solutions such as the naïve Bayes classifier [39], more sophisticated approaches to resolving such issues of false positive assignments could include examining BLAST-based sequence similarity matches to taxa beyond the first match reported. For example, one source of false positives is reads that map to highly conserved regions of sequences. Such reads are likely to possess many sequence similarity matches with the same BLAST score cut-offs. Through considering abundant taxa identified through mappings to other reads, it is possible to devise an algorithm that selects the most likely match, from a list of matches sharing the same score. In the next section, we explore these issues further through comparing the performance of 16S- and mRNA-derived reads to assess diversity within and between samples.

### Consistency of diversity analyses between 16S rRNA and mRNA datasets

We assessed species diversity for each sample based on putative mRNA reads and compared them to species representations derived from filtered 16S rRNA reads obtained in our pipeline (see ‘Methods’). Four ecological biodiversity indices were employed: three based on diversity measures (Shannon diversity index, Simpson index and Fisher’s alpha) and the Chao1 richness index (Table 2). Amongst the diversity indices, the Shannon and Fisher’s alpha are broadly consistent with the exception of the two individual cecal-derived samples, which Fisher’s alpha suggest are less diverse than the Kimchi dataset. Conversely, the Simpson index rates the mouse cecal-flush sample as the most diverse. However, in general, across samples and consistent with the large number of species with broad transcript representation, the deep-sea dataset was found to be the most diverse and rich with the results based on mRNA reads (5.01 and 4408 for Shannon and Chao1 indices, respectively). Conversely, the kimchi dataset was the least diverse and rich, likely due to the dominance of a few taxa (1.69 and 634). Noteworthy, the permafrost dataset appeared the least diverse microbiome based on the Chao1 index but not for any diversity based index. This is likely due to the small number of annotatable reads associated with this dataset.

**Table 2:** Diversity analysis within mice samples and between five samples

Sample name	Shannon index (mRNA)	Simpson index (mRNA)	Fisher’s alpha (mRNA)	Shannon index (16S rRNA)	Chao1 index (mRNA)	Chao1 index (16S rRNA)
Mouse cecal wall	3.83	16.51	23.26	2.00	1162	283
Mouse cecal flush	4.43	43.34	30.52	2.57	1055	411
Mouse combined	4.51	17.14	167.33	2.48	1709	523
Cow rumen	4.14	21.79	140.67	4.15	1461	1042
Kimchi	1.69	3.27	56.07	2.91	634	615
Deep sea	5.01	35.75	481.29	5.02	4408	4565
Permafrost	3.82	10.98	24.31	4.5	295	348

Comparing between sequence types, we find broad consistency between the results for the 16S rRNA and mRNA based analyses, with the exception of the mouse samples. For the latter datasets, while the 16S rRNA gene analyses yielded lower diversity metrics for the mouse datasets (reflecting the limited number of taxa associated with the altered Schaedler flora (ASF) used to inoculate germ free mice), the mRNA-based analyses yielded comparatively higher diversity metrics. This is likely due to the challenge of mapping the putative mRNA reads in these datasets to their correct taxa in the absence of ASF reference genomes used for mapping. Instead, reads appear to have been assigned to multiple closely related taxa. We note for example that this does not arise for the kimchi dataset for which there is good representation of reference genomes. Although the 16S rRNA- and mRNA-based diversity and richness analyses are largely consistent, excluding the permafrost dataset, we find that from 56 % (kimchi) to 81 % (mouse) of genera identified from 16S rRNA reads overlap with reads of mRNA origin (Additional File 4). At the same time, we also note many genera predicted by the mRNA reads compared to the 16S rRNA reads, with the former predicting from 83 % (kimchi) to 478 % (deep sea) additional genera. Such additional predictions likely arises from a combination of the lack of a complete set of reference datasets for both mRNA or 16S rRNA reads, as well as mispredictions from the taxonomic annotation pipeline as noted above. Nevertheless, given the consistency in diversity and richness metrics between sequence types for the cow rumen, kimchi and deep-sea datasets, these results suggest that even short-read data derived from mRNA can reveal significant taxonomic differences that reflect genuine differences in habitat.

In the following sections, we show how this information may be leveraged to identify distinct taxonomic contributions towards biochemical activities within a microbiome.

### Functional interrogation of metatranscriptome datasets reveals a conserved core of essential metabolic functions supplemented with habitat-specific pathways

**Fig. 3:**
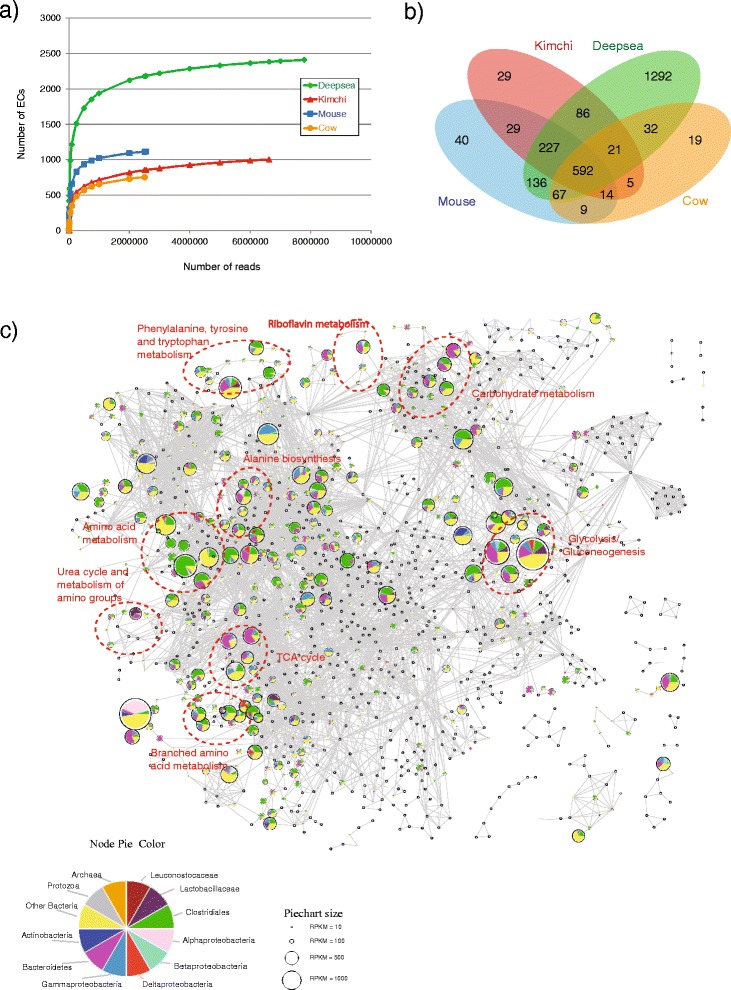
Metabolic composition of five metatranscriptomic datasets. **a** Rarefaction analysis indicating the number of unique enzymes (as defined by enzyme classification numbers) captured by increasing numbers of putative mRNA reads generated. **b** Overlap of enzyme complements across four datasets reveals a common core of 592 enzymes. **c** Global metabolic network indicating taxonomic representation of metabolic activities within the combined mouse dataset. Pie charts indicate the relative proportion of each taxon, size of pie chart indicates relative expression (see key). Indicated are specific metabolic pathways

A major challenge in metatranscriptomic studies is determining the depth of sequencing required to adequately capture the functional capacity of a microbiome (i.e. ‘how much sequencing is enough?’). Focusing on metabolism, we performed a rarefaction analysis of enzyme annotations captured by increasing numbers of reads within the five datasets. As expected, all five datasets revealed an asymptotic relationship between number of reads generated and enzymes (as defined by distinct Enzyme Classification (EC) numbers—Fig. 3a). For the two largest datasets, kimchi and deep sea, we find that for ~4 million putative mRNA reads, the rate of new enzyme discovery is 30 and 45 per million reads, respectively. Given a current expected yield of 20 % reads of putative mRNA origin, our analysis suggests that the generation of ~20 million reads for a microbiome provides a reasonable compromise between sequencing costs and enzyme discovery. However, such decisions should also assess additional factors such as mirobiome complexity; we note that the deep-sea dataset contained the greatest metabolic capacity. Due to the relatively low number of putative mRNA reads (~14,300) suggesting only a limited sampling of its metabolic capacity, the permafrost dataset was excluded from subsequent analyses.

Comparisons across the four datasets revealed a common core of 592 enzymes (Fig. 3b). These core enzymes were significantly associated (hypergeometric test, *p* value <0.01) with 22 pathways, as defined by the Kyoto Encylcopedia of Genes and Genomes (KEGG—Table 3) [40]. These pathways represent core metabolic functions including carbohydrate and energy metabolism (7 pathways), amino acid metabolism (5 pathways) and nucleotide metabolism (2 pathways). It is suggested that future studies consider using enzymes involved in these pathways as a benchmark to assess the quality and coverage of their datasets. For example, within the permafrost dataset, of 152 defined enzymes, only 93 (15.8 % of our defined core) are present. These include 5 of 40 (12.5 %) core enzymes associated with nucleotide metabolism, 13 of 84 (15.5 %) core enzymes associated with amino acid metabolism, 10 of 66 (15.2 %) core enzymes associated with carbohydrate metabolism, 22 of 119 (18.5 %) core enzymes associated with multiple pathways and 25 of 146 (17.1 %) core enzymes that were not assigned into a KEGG defined pathway. Hence, it appears that enzymes in core pathways missing in the permafrost dataset are relatively evenly distributed across functional categories, reflective of lower coverage rather than microbiome bias.

**Table 3:** Pathways significantly enriched in ‘core’ microbiome enzymes

Pathway name	Pathway class^a^	*p* value^b^	Core enzymes in pathway	Total enzymes in pathway
Aminoacyl-tRNA biosynthesis	O	3.57E-08	22	32
Purine metabolism	NT	1.49E-06	44	100
Peptidoglycan biosynthesis	G	4.30E-06	12	15
Glycolysis/gluconeogenesis	C	2.89E-05	23	45
Alanine, aspartate and glutamate metabolism	AA	4.24E-05	22	43
Valine, leucine and isoleucine biosynthesis	AA	8.75E-05	12	18
Pyrimidine metabolism	NT	2.89E-04	27	63
Phenylalanine, tyrosine and tryptophan biosynthesis	AA	6.27E-04	15	29
Pentose phosphate pathway	C	7.05E-04	17	35
Carbon fixation pathways in prokaryotes	E	2.22E-03	17	38
One carbon pool by folate	CO	3.21E-03	12	24
Lysine biosynthesis	AA	3.32E-03	13	27
Pyruvate metabolism	C	3.37E-03	24	62
Fatty acid biosynthesis	L	3.88E-03	9	16
Citrate cycle (TCA cycle)	C	4.76E-03	11	22
Amino sugar and nucleotide sugar metabolism	C	5.66E-03	30	85
Oxidative phosphorylation	E	8.48E-03	7	12
Drug metabolism—other enzymes	X	2.34E-02	9	20
Cysteine and methionine metabolism	AA	2.55E-02	21	61
Polyketide sugar unit biosynthesis	T	2.74E-02	4	6
Streptomycin biosynthesis	S	3.48E-02	8	18
Folate biosynthesis	CO	3.61E-02	7	15

^a^Defined according to KEGG. *AA* amino acid, *C* carbohydrate, *CO* co-factor, *E* energy, *G* glycan, *L* lipid, *NT* nucleotode, *O* other, *S* secondary metabolites, *T* terpenoids, *X* xenobiotics

^b^Here, we used the hypergeometric test to examine enrichment of pathways compared to all KEGG defined pathways

In addition to the core enzymes, we also identified the unique expression of enzymes providing habitat-specific biochemical functions (Additional files 5 and 6). For example, the deep-sea dataset includes enzymes involved in phosphonate metabolism, a significant component of organic phosphorous in the marine environment [41]. Similarly, the glucosyltransferase, levansucrase (EC: 2.4.1.10), was uniquely associated with the kimchi dataset. Levansucrase is involved in the synthesis of glucose polymers and was previously isolated and characterized from a key member of the kimchi community, *Leuconostoc mesenteroides* [42]. Unique to the cow rumen dataset were pectate di- and tri-saccharide lyases, reflecting the presence of pectin in animal feed and thought to be responsible for supporting the growth of *Trepnonema* sp*.* [43].

In the next section, we combine the taxonomic and metabolic annotation data to examine the contributions of specific taxa to biochemical activities in the sampled microbiomes.

### Integration of taxonomic and functional annotations: I. Metabolic networks

While previous microbiome studies have associated shifts in taxonomic distributions and/or biochemical functions with disease states or other evolving habitats, such as the process of fermentation [9, 44, 45], our understanding of the contribution of specific taxa to these functions is limited. In the previous sections, we demonstrated the capacity of short sequence reads associated with metatranscriptomic datasets to provide both taxonomic and functional insights. In the following sections, we show how the integration of such information can be used to derive a more complete understanding of how different taxa contribute towards the biochemical activities of a microbiome.

**Fig. 4:**
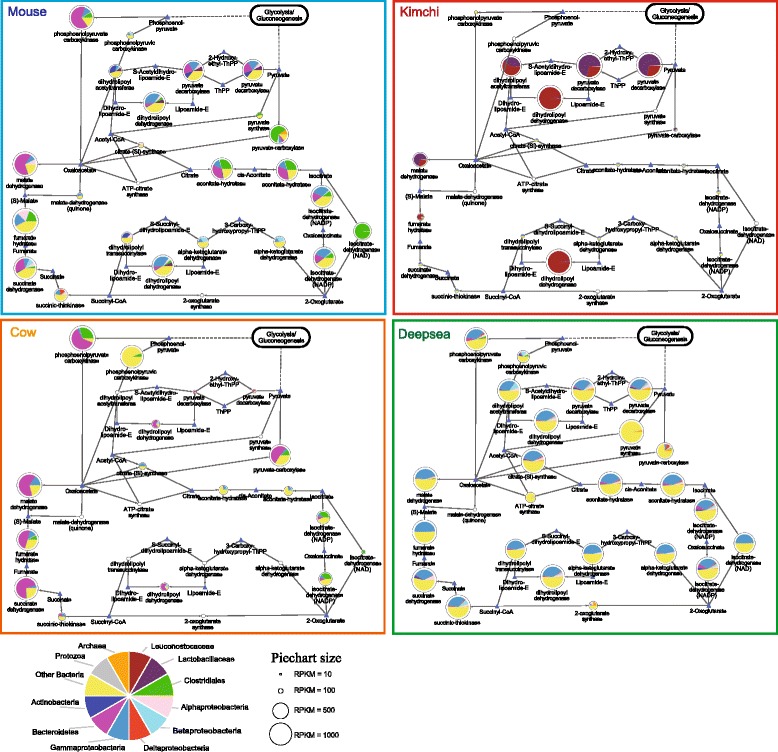
Detailed views of taxonomic contributions to specific components of the tricarboxylic acid (TCA) cycle for four metatranscriptomic datasets. Each schematic indicates the taxonomic representation of enzymatic activities involved in the TCA cycle for four metatranscriptome datasets: mouse, kimchi, cow and deep sea. Pie charts indicate enzymes, with coloured sectors indicating the relative proportion of each taxon, size of pie chart indicates relative expression (see key). *Small triangles* indicate substrates with links indicating enzyme-substrate relationships

Given the limits of taxonomic resolution identified above, we chose to divide reads into twelve taxonomic categories including archaea and protozoa. From these assignments, we constructed a global metabolic network graph in which nodes, representing enzymes, are linked through shared substrates. Each node is depicted as a pie chart in which the relative contribution of each taxon is represented as a slice (Fig. 3c and Additional files 7, 8 and 9). These global views of metabolism enable the identification of biochemically related enzymes sharing similar taxonomic profiles. For example, for the mouse dataset, reads originating from Clostridiales dominate several amino acid pathways as well as parts of the glycolytic pathway (Additional file 10). Pathways such as pyruvate metabolism, the tricarboxylic acid (TCA) cycle and alanine, aspartate and glutamate metabolism feature larger contributions from other taxa such as Gammaproteobacteria and Bacteroides (Fig. 4 and Additional file 11).

Comparisons across samples further reveal that as noted above, many pathways are conserved but the taxa responsible for these pathways as well as their relative expression are not conserved (Fig. 3c, Fig. 4 and Additional files 7, 8, 9, 10 and 11). For example within the TCA cycle, relative to the cow rumen dataset, the other three samples feature high expression of enzymes that together comprise the pyruvate dehydrogenase complex involved in anaerobic fermentation, e.g. dihydrolipoyl acetyltransferase (EC: 2.3.1.12), dihydrolipoyl dehydrogenase (EC: 1.8.1.4) and pyruvate decarboxylase (EC: 1.2.4.1). However, whereas Actinobacteria, Bacteroides and Gammaproteobacteria contribute significant reads to these enzymes in the mouse dataset, these enzymes are represented largely by Gammaproteobacteria in the deep-sea dataset and by Leuconostocaceae and Lactobacillaceae in the kimchi dataset. Furthermore, within a sample, we identify pathway sections that feature distinct taxonomic profiles. For example in the mouse intestinal dataset, Clostridiales contribute significantly to pyruvate carboxylase (EC: 6.4.1.1) as well as members of the TCA cycle. Also, noteworthy is the relatively high expression of phosphoenolpyruvate carboxykinase (EC: 4.1.1.49) in the mouse intestinal and cow rumen datasets. Previously associated with *Ruminococcus flavefaciens*, a Clostridiales bacterium found in the rumen [46] and *Bacteroides fragilis* found in the human gut [47], this enzyme is believed to be involved in the fermentation of cellulose to succinate in the rumen and catalyses phosphoenolpyruvate to oxaloacetate with the concomitant formation of ATP in human gut, may act as a ‘feeder’ reaction for carbon from the TCA cycle to drive various biosynthetic and oxidative processes such as gluconeogenesis and serine synthesis [48].

Focusing on glycolysis/gluconeogenesis (Additional file 10), as for the TCA cycle, we found that taxonomic groups that dominate the entire datasets also dominate specific enzyme activities. However again, sections of the pathway can be dominated by specific taxa. For example, aldose 1-epimerase (EC: 5.1.3.3) in the cow rumen and l-lactate dehydrogenase (EC: 1.1.1.27) in kimchi are predominantly expressed by Bacterioidetes and Lactobacillaceae, respectively. Further, even apparently minor taxa appear to provide specific functionality, suggestive of keystone roles within the community. For example, in the mouse intestinal dataset, both alcohol dehydrogenase (EC: 1.1.1.2) and aldose 1-epimerase (EC: 5.1.3.3) are predominantly expressed by Lactobacillaceae despite representing only 1.9 % of putative mRNA reads. As a final example of taxonomic contributions to metabolic functionality, we find that for the mouse intestinal dataset, Bacterioidetes and Gammaproteobacteria tend to dominate aspartate metabolism, while Closteridiales dominate glutamate metabolism (Additional File 11). As for the TCA cycle, while the majority of enzymes are well expressed in the mouse intestinal dataset, for the Kimchi dataset, expressions of genes within these pathways are more heterogeneous. This raises an important caveat, notably that the ability to map reads to the enzymes is dependent on the availability of suitable sequences in the reference databases. Hence, an inability to assign reads to asparagine synthase (EC 6.3.5.4) in the kimchi dataset may be due to the inability of sequence searches to map reads from the orthologous genes in the kimchi microbiome to known examples of this enzyme in the reference database.

### Integration of taxonomic and functional annotations provides molecular level insights into the biochemical contributions of individual taxa: II. Protein-protein interaction networks

**Fig. 5:**
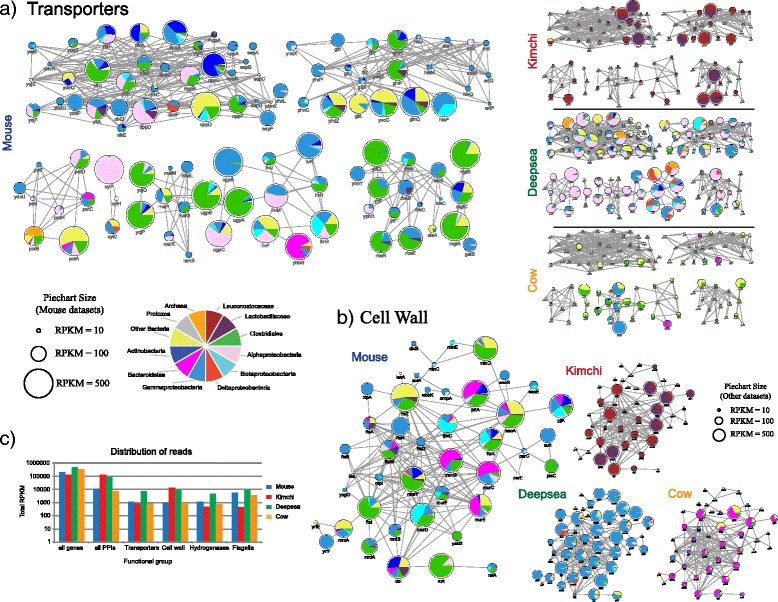
Taxonomic contributions to functional modules defined through protein-interaction networks. **a** ABC transporters and (**b**) cell wall biogenesis and cell division. Protein interactions were obtained from a previously generated network of functional interactions for *E. coli* [49]. Pie charts indicate the relative proportion of each taxon, size of pie chart indicates relative expression (see key). **c** Relative representation of specific functional groups across the four well sampled datasets

Beyond metabolic pathways, the provision of protein-protein interaction (PPI) networks offers additional opportunities to explore taxa-specific contributions to biochemical processes. Here, we integrate taxonomic information with a PPI network previously constructed for *Escherichia coli* [49]. The ABC transporter superfamily is a collection of transporters typically comprised of an extracellular substrate binding subunit, an intracellular ATP-binding subunit and a membrane incorporated permease. Across the different datasets, we see distinct signatures of subunit expression and taxonomic contributions (Fig. 5a). For example, while many members of this family are well expressed in the mouse intestine, expression within the kimchi dataset is largely limited to putrescine-ornithine transport (potA-D), oligopeptide transport (oppA-D and F), ribose transport (rbsB-DK and R) and members or glutamine, histidine and arginine transport (e.g. glnH, glnP, hisP and argT). Similarly, in cow rumen, only a subset of transporters were well represented; these included xylF-H (xylose), malE-GK (maltose) and ugpA-CE (glycerol-3-phosphate).

In the deep-sea microbiome dataset, many transporter components were associated with alphaproteobacteria, although leucine, isoleucine and valine transport components (livF-HJK and M) were broadly represented across phyla. In the mouse dataset, alphaproteobacteria were also the main contributors of transporters including dipeptide ABC transporter (dppBD), glutathione ABC transporter (yliABC), leucine ABC transporter (LivMF), glycerol-3-phosphate ABC transporter (ugpC) and xlycose ABC transporter (xylFH). xylF was largely represented by clostridiales and ‘other bacteria’ in the cow rumen dataset, suggesting that the contribution of alphaproteobacteria in the mouse data does not reflect annotation bias. The mouse intestine samples also display Gammaproteobacteria and Actinobacteria contributions to transporter components. Finally, the lack of Bacteroidetes representation in transporter components may reflect the reduced complement of these genes previously noted for members of this phylum [50, 51].

Many genes involved in cell wall biogenesis and cell division were expressed within all datasets (Fig. 5b). Of these, secA, prlA(secY) and ftsZ were the most highly expressed in each dataset. SecA mediate critical roles in protein translocation, and ftsZ is involved in organizing the initial stages of cell division. Within the mouse datasets, few reads from Bacteroidetes were assigned to ftsZ, suggesting that the ortholog(s) within this taxon display significant divergence from their *E. coli* counterpart. For example, the conserved C-terminus of *E. coli* ftsZ is absent in Bacteroidetes [52]. Genes encoding proteins involved in later steps of cell division (e.g. ftsN, ftsB, ftsQ and zipA) were largely restricted to representation by Gammaproteobacteria, suggesting these sequences are highly specialized within this taxon. Genes involved in the synthesis of cell wall components (e.g. mur and mrd) were well represented across the datasets, with the mouse and kimchi datasets featuring clear patterns of taxonomic contributions. For example, within the mouse dataset, murCEG were well represented by Bacteroidetes, while for the kimchi dataset, mrdA and mrdB were largely represented by the Lacteobacillaceae, potentially representing altered cell wall composition in these taxa.

Unlike cell wall biogenesis and cell division, genes involved in flagella assembly, chemotaxis and hydogenases were poorly represented in the four datasets (Additional files 12 and 13). For example, both cow and kimchi datasets lacked significant expression of many flagella and chemotaxis genes reflecting an absence of flagella in many of the major taxa in these microbiomes (e.g. *Lactobacillus* spp*.* and *Leuconostoc* spp*.* in kimchi). Indeed, for kimchi, expression was largely limited to flgJ, a peptidoglycan hydrolase required for flagella formation and likely reflects significant local sequence similarity with other proteins such as *N*-acetylmuramoyl-l-alanine amidase from *L. sakei* which shares a conserved, ~200 residue lysine motif with flgJ. In the mouse, we noted little representation from Bacteriodetes, with most expression dominated by Closteridiales. As noted above, the restriction of certain components to Gammaproteobacteria may reflect their relative sequence diversity and/or specialized functions. Finally, we note that four genes were dominated by representation from the Alphaproteobacteria: motA, mbhA, cheY and flip. Such abundance may at least in part be due to variable copy numbers of these genes in this taxon, for example, cheY is present in six copies in the *Rhodobacter sphaeroides* genome [53].

Finally, we also explored the expression and taxonomic representation of genes involved in NADH dehydrogenase and hydrogenase complexes (Additional file 13). As for flagella assembly and chemotaxis, many components were not represented within the four samples. For example, 16 of the 50 genes that comprise these complexes lack expression in the kimchi, cow rumen and deep-sea datasets. Indeed, within the kimchi dataset, only tpiA, dps and iscS are well represented. This is likely related to local sequence conservation between the Fe-binding motif of dps and the cysteine desulfurization and conservative C-terminal of iscS, resulting in misannotations. Curiously, while both the cow and deep-sea datasets feature relatively homogenous patterns of taxonomic representation in their respective NADH dehydrogenase subunits, those in the mouse dataset appear largely incongruent.

## Conclusions

In this study, we present a standard bioinformatics pipeline to process, annotate and analyse metatranscriptomic datasets. Applied to five disparate metatranscriptomic datasets (mouse cecum, cow rumen, kimchi, deep sea and permafrost), this pipeline captures both common and microbiome-specific taxonomic and functional signatures. In general, each microbiome is dominated by members of four bacterial phyla (*Firmicutes*, *Proteobacteria*, *Bacteroidetes*, *Actinobacteria*) and one archaeal phylum; however, each microbiome features distinct differences in the relative representation at higher phylogenetic levels (i.e. families and genera). Diversity analyses reveals that mRNA taxonomic representation is broadly congruent with 16S taxonomic representation, with the proviso that a lack of suitable reference genomes can result in mRNA datasets overestimating diversity. Comparisons of microbiome metabolic capacities revealed a core of 592 enzymes common to the four well-sampled microbiomes (i.e. ignoring permafrost), largely associated with housekeeping functions such as carbohydrate, amino acid and nucleotide metabolism. While the concept of ‘core’ bacterial functions have previously been described for individual taxa (e.g. [54]), this concept has yet to be explored from a metatranscriptomic viewpoint. Such conserved pathways provide a valuable benchmark to assess the quality and coverage of metatranscriptomic datasets. Furthermore, we identified microbiome-specific enzymes reflecting distinct differences in habitat. We choose to compare mouse cecal flush and cecal wall samples to determine whether gene expression is substantially different in the wall-adherent compared to luminal microbiome. Analyses with three established tools identified only a limited set of differentially expressed genes between the cecal wall and cecal flush samples. However, a gene set enrichment approach applying a fold-change metric identified several pathways of differentially expressed genes at these two locations suggesting that biogeographical differences require additional study in mammalian gut microbiomes. Finally, integration of phylogenetic and functional annotations within a systems context provides a powerful route to identify the relationship between taxonomic representation within a microbiome and their contribution to biochemical activities; while dominant taxa appear broadly represented across biochemical pathways, key contributions may be performed by a more limited set of less abundant taxa.

## Methods

### Metatranscriptomic datasets and initial processing

Publically available metatranscriptomic datasets were obtained from the National Center for Biotechnology Information (NCBI) sequence read archive (SRA, http://www.ncbi.nlm.nih.gov/sra; [55]). These include samples consisting of:

1. Thirty million pairs of 76 bp reads derived from the luminal contains of the cecal wall and cecal flush of four non-obese diabetic (NOD). Mice were born and maintained in germ-free environment and subsequently colonized with altered Schaedler flora (ASF), a defined community of eight known bacterial species: *L. acidophilus*, *L. murinus*, *B. distasonis*, *M. schaedleri*, *Eubacterium plexicaudatum*, an uncharacterized fusiform bacterium and two uncharacterized clostridium species (12 samples total—SRX134832-40, SRX134842 and SRX134844-45; [11]).
2. Thirty-five million 101 bp reads derived from a 29 day fermentation of kimchi (SRX128705; [9]).
3. Fourteen million pairs of 100 bp reads derived from the rumen of a Holstein dairy cow fed a fat-supplemented diet (SRX196213; [8])
4. One hundred three million pairs of 100 bp reads derived from the Guaymas Basin hydrothermal vent (SRX1347659; [10]).
5. One hundred thirty-one million pairs of 150 bp reads derived from permafrost soil (SRX119222).

For each sequence, low quality segments (Phred score <15 [56]) were trimmed using an in-house script and reads <50 bp discarded. Next adaptor contaminants were filtered using cross-match (http://www.phrap.org) with parameters minmatch = 10 and minscore = 20. In addition, due to the large number of low quality reads in the permafrost sample, we applied an in-house script to remove those containing 10 consecutive N’s and/or X’s. rRNA reads were identified and removed by first applying BWA [13], with a bitscore cut-off of >50, against a database of rRNA genes collated from the SILVA, Greengenes and NCBI databases [57–59]. Additional reads of rRNA and tRNA origin were identified using the Infernal software [60] with the Rfam database as a reference [61]. For mouse, cow and kimchi datasets, reads of murine, bovine and plant origin were identified and removed through BLAT searches (bitscore cutoff >50) against the mouse genome and transcriptome (build GRCm38 downloaded from Ensemble [62]), the cow genome and transcriptome (build 6.1 downloaded from NCBI [63]) and a set of 25 plant genomes and 274 plant transciptomes obtained from the PlantGDB database [64], respectively.

### Assembly and annotation

To increase efficiency of annotation, putative mRNA reads were assembled by the de novo assembly package Trinity [20]. Reads were mapped back to contigs using the Bowtie alignment tool [65]. Sequence annotation was performed using a tiered set of sequence alignment tools: BWA [13], BLAT [21] and BLAST [66]. BWA and BLAT alignments were performed using default parameters against a reference database of 4443 prokaryotic genomes (including 1918 plasmid, 152 archaeal and 2373 bacterial genomes) downloaded from the NCBI (February, 2013). For BWA, this translates to no more than two mismatches over the entire alignment, although we note that previous studies suggest that different parameter settings result in highly similar output [67]. Reads that could not be aligned through BWA and BLAT were subject to BLASTX sequence similarity searches against the protein non-redundant database obtained from the NCBI (February, 2013). Two thresholds were used: (1) for reads shorter than 100 nts, read alignments were considered if sequence identity was ≥85 % over >65 % of the read length; and (2) for reads longer than 100 nts, we applied a more stringent bit score cut-off of 60. Enzyme annotations for genes and proteins matching sequence reads was performed using: (1) DETECT enzyme prediction tool [68] and (2) BLASTP sequence similarity searches against a set of enzymes curated by UniProt (*e*-value <1e-10) [69]. Where DETECT and BLASTP annotations conflicted, DETECT predictions were assumed to be more reliable [68]. Transcript expression was normalized using reads per kilobase of transcript per million mapped reads (RPKM [70]).

### Analysis of differential expression

Differential expression analyses (genes and metabolic pathways) were focused on the seven mouse samples (three cecal flush and four cecal wall) that had been prepared using the same RNA extraction treatment (RNeasy—Qiagen Inc., Valencia, CA): SRX134832, SRX134834, SRX134835, SRX134837, SRX134838, SRX134840 and SRX134842. The performance of three algorithms were examined: edgeR [25], DEseq2 [24] and ALDEx2 [26]. Both edgeR and DESeq2 were originally developed for microarray analyses and have recently been updated for RNA-Seq data (edgeR v2.14 and DEseq2, v2.14), while ALDEx2 (version 1.0.0) was developed specifically for RNA-Seq datasets. Initial analyses identified high variation within and between the cecal wall and cecal flush samples as measured by the biological coefficient of variation, a measure used to assess differential expression in RNA-Seq experiments [71] and calculated as:

$$ {\mathrm{CV}}^2\left({y}_{\mathrm{gi}}\right) = \mathrm{v}\mathrm{a}\mathrm{r}\ \left(\ {y}_{\mathrm{gi}}\right)\ /\ {\mu_{\mathrm{gi}}}^2 = 1/{\mu}_{\mathrm{gi}} + {\varphi}_g $$

Where 1/*μ*_gi_ is the squared CV for the Poisson distribution and *φ*_*g*_ is the squared CV of the unobserved expression values. We therefore estimated the variation per pairwise replicates using the Kruskal-Wallis test and removed three samples displaying extreme variation (*p* < 0.001): SRX134837, SRX134838 and SRX134842. In addition to the algorithms applied above, we also applied a pathway enrichment analysis of genes displaying at least a fivefold change of expression (defined by the average expression for the four remaining samples (two cecal flush and two cecal wall)) [27, 28]. Here, we applied the hypergeometric test by computing two-tailed *p* values for differentially expressed genes for reference pathway sets obtained from the KEGG [17].

### Analysis of microbial composition and diversity

Taxonomic classifications of transcripts were derived from the tiered set of read annotation searches with reference to the NCBI taxonomy database. For the comparative tree based analysis presented in Fig. 2b, we included only those genera represented by greater than 100 reads across all five microbiomes (966 genera total). To normalize genus representation across microbiomes, each genus was divided by the average number of reads assigned to each of the 966 genera and only genera with normalized read values in excess of 10 were visualized. Visualization was performed using MEGAN5 [72] in conjunction with the Interactive Tree of Life (iTOL) software [73] to modify and annotate the resulting phylogenetic tree.

Three measures were applied to measure sample diversity: Shannon entropy [74], Fisher’s alpha diversity index [75] and Simpson diversity [75]. In addition, we also measured taxonomic ‘richness’, using the richness index—Chao1 [76]. To reduce the incidence of false positives and consistent with previous studies (e.g. [77–79]), only species represented by at least 100 reads were included in these analyses. The relative abundance of species was normalized by the average read count for each sample. To compare diversity metrics obtained with putative mRNA sequences to those obtained with 16S rRNA sequences, we mapped putative 16S rRNA reads identified in the samples by our pipeline to 16S rRNA sequences retrieved from the SILVA database [59] using BWA. These sequences were then clustered into ‘species’ at 97 % identity using CD-HIT [80]. For the Shannon entropy, the non-parametric method was applied:

$$ {H}_{\mathrm{sh}}=-{\displaystyle \sum_{i=1}^S}{p}_i \log {p}_i $$

Where *S* is the number of species and *p*_*i*_ is the relative abundance of species *i* (defined by the number of reads associated with that species). Fisher’s alpha diversity index was calculated as:

$$ S=a\times \ln \left(1+n/a\right) $$

Where *S* is number of taxa, *n* is number of individuals and *a* is the Fisher’s alpha. Simpson diversity was calculated by:

$$ D={\displaystyle \sum \left({\left({n}_i/n\right)}^2\right)} $$

Where *n*_*i*_ is number of individuals of taxon i. Finally, Chao1 scores were calculated by:

$$ {\widehat{s}}_{\mathrm{chao}1}={S}_{\mathrm{obs}}+\frac{n_1^2}{2{n}_2} $$

Where *S*_obs_ is the total number of species observed in all samples, *n*_1_ is the number of singletons (species captured once) and *n*_2_ is the number of doubletons (species captured twice). Diversity indices based on these values were calculated using EstimateS v 9.1 [81] using 100 bootstrap replicates.

### Network visualization

Metabolic networks were constructed as previously described [54]: enzymes (EC numbers) are represented as nodes and substrates connecting two enzymes are represented as edges in the network. Enzyme-substrate relationships were inferred from KEGG [40]. Protein-protein interaction (PPI) networks were constructed by homology mapping of *E. coli* homologs of identified bacterial transcripts using BLAST sequence similarity searches (*E*-value <1e^-10^) and layering expression data onto a previously generated network of PPIs for *E. coli* [49]. To compare expression across microbiomes, RPKM values of each enzyme/*E. coli* homolog was normalized by employing the min-max scaling method. Networks were visualized using Cytoscape version 3.2.1 [82] and iPath [83].

## Additional files

Additional file 1:
**Summary of metatranscriptome processing, assembly and annotation steps.** This table provides summary statistics for read processing steps applied to each dataset. (XLSX 16 kb) Additional file 2:
**Transcripts displaying significant (** ***p*** **< 0.05) differences in expression between mouse cecal wall and cecal flush datasets as determined by three statistical programs (edgeR, DEseq2 and ALDEx2).** This table lists all transcripts displaying significant differences in expression between the two types of mouse samples. (XLSX 17 kb) Additional file 3:
**Transcripts displaying greater than five-fold difference in expression between cecal wall and cecal flush samples.** This table lists all transcripts displaying large differences in expression between the two mouse datasets. (XLSX 56 kb) Additional file 4:
**Venn diagram illustrating overlap of genera defined by putative 16S rRNA and mRNA reads for five metatranscriptomic datasets.** Numbers indicate the number of genera defined by each type of sequence data. (PDF 112 kb) Additional file 5:
**Enzyme expression for five metatranscriptomic datasets.** This table lists the expression (in terms of RPKM) for each enzyme annotated in our datasets. (XLSX 226 kb) Additional file 6:
**iPath representation of common and unique enzymes in a global metabolism map from KEGG.** The global metabolic map was generated using the online iPath tool [83] with reactions coloured according to their presence in different metatranscriptomic datasets (see inset key). (PDF 1815 kb) Additional file 7:
**Global metabolic network indicating taxonomic representation of metabolic activities within the cow rumen dataset.** Global metabolic network indicating taxonomic representation of metabolic activities within the cow rumen dataset. Pie charts indicate the relative proportion of each taxon, size of pie chart indicates relative expression (see key). Indicated are specific metabolic pathways. (PDF 1162 kb) Additional file 8:
**Global metabolic network indicating taxonomic representation of metabolic activities within the kimchi.** Global metabolic network indicating taxonomic representation of metabolic activities within the kimchi dataset. Pie charts indicate the relative proportion of each taxon, size of pie chart indicates relative expression (see key). Indicated are specific metabolic pathways. (PDF 1352 kb) Additional file 9:
**Global metabolic network indicating taxonomic representation of metabolic activities within the deepsea dataset.** Global metabolic network indicating taxonomic representation of metabolic activities within the deepsea dataset. Pie charts indicate the relative proportion of each taxon, size of pie chart indicates relative expression (see key). Indicated are specific metabolic pathways. (PDF 2066 kb) Additional file 10:
**Detailed views of taxonomic contributions to specific components of the glycolysis/gluconeogenesis pathway for four metatranscriptomic datasets.** Each schematic indicates the taxonomic representation of enzymatic activities involved in the glycolysis/gluconeogenesis pathways for four metatranscriptome datasets: mouse, kimchi, cow and deepsea. Pie charts indicate enzymes, with coloured sectors indicating the relative proportion of each taxon, size of pie chart indicates relative expression (see key). Small triangles indicate substrates with links indicating enzyme-substrate relationships. (PDF 635 kb) Additional file 11:
**Detailed views of taxonomic contributions to specific components of the alanine, aspartate and glutamate pathways for four metatranscriptomic datasets.** Each schematic indicates the taxonomic representation of enzymatic activities involved in the alanine, aspartate and glutamate pathways for four metatranscriptome datasets: mouse, kimchi, cow and deep sea. Pie charts indicate enzymes, with coloured sectors indicating the relative proportion of each taxon, size of pie chart indicates relative expression (see key). Small triangles indicate substrates with links indicating enzyme-substrate relationships. (PDF 658 kb) Additional file 12:
**Taxonomic contributions to flagella biosynthesis and chemotaxis modules as defined through protein-interaction networks for the four well sampled datasets.** Each network indicates the taxonomic representation of components of flagella biosynthesis and chemotaxis modules as defined through protein-interaction networks for four metatranscriptome datasets: mouse, kimchi, cow and deepsea. Pie charts indicate genes, with coloured sectors indicating the relative contribution to gene expression for each taxon, size of pie chart indicates relative expression (see key). (PDF 202 kb) Additional file 13:
**Taxonomic contributions to select hydrogenase modules as defined through protein-interaction networks for the four well sampled datasets.** Each network indicates the taxonomic representation of components of hydrogenases as defined through protein-interaction networks for four metatranscriptome datasets: mouse, kimchi, cow and deep sea. Pie charts indicate genes, with coloured sectors indicating the relative contribution to gene expression for each taxon, size of pie chart indicates relative expression (see key). (PDF 190 kb)
