# Supplementary material for: Metatranscriptomic analysis of diverse microbial communities reveals core metabolic pathways and microbiome-specific functionality
Source: Microbiome. 2016 Jan 12;4:2. doi: 10.1186/s40168-015-0146-x (PMC4710996; doi:10.1186/s40168-015-0146-x)

Additional File 4 Venn diagrams showing overlap in number of genera predicted from reads of mRNA and 16S rRNA origin for five metatranscriptomic samples

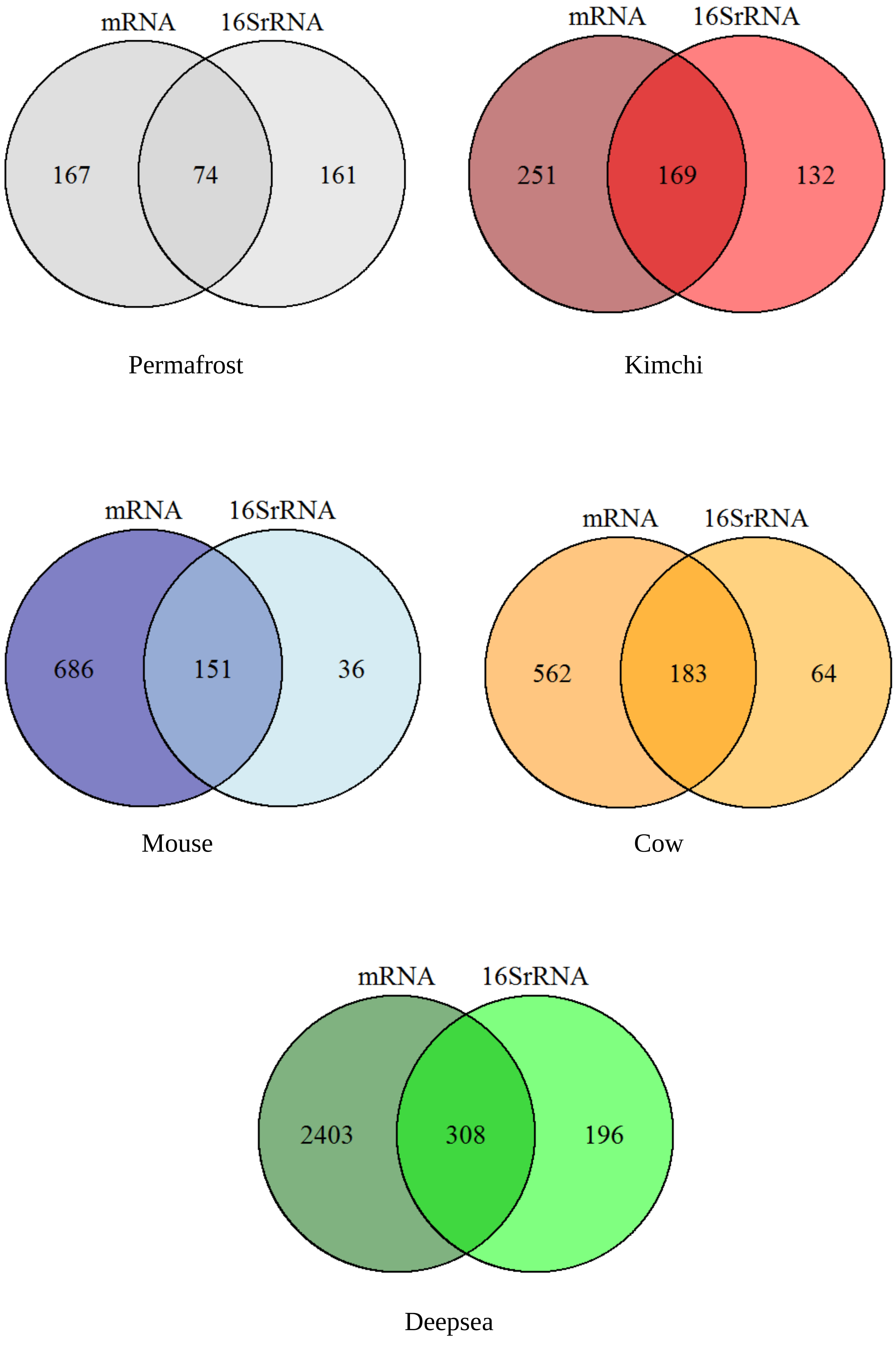

Supplement: Additional file 4: — Venn diagram illustrating overlap of genera defined by putative 16S rRNA and mRNA reads for five metatranscriptomic datasets. Numbers indicate the number of genera defined by each type of sequence data. (PDF 112 kb) [file 40168_2015_146_MOESM4_ESM.pdf]
