# Supplementary figures and images for: Metatranscriptomic analysis of diverse microbial communities reveals core metabolic pathways and microbiome-specific functionality
Source: Microbiome. 2016 Jan 12;4:2. doi: 10.1186/s40168-015-0146-x (PMC4710996; doi:10.1186/s40168-015-0146-x)

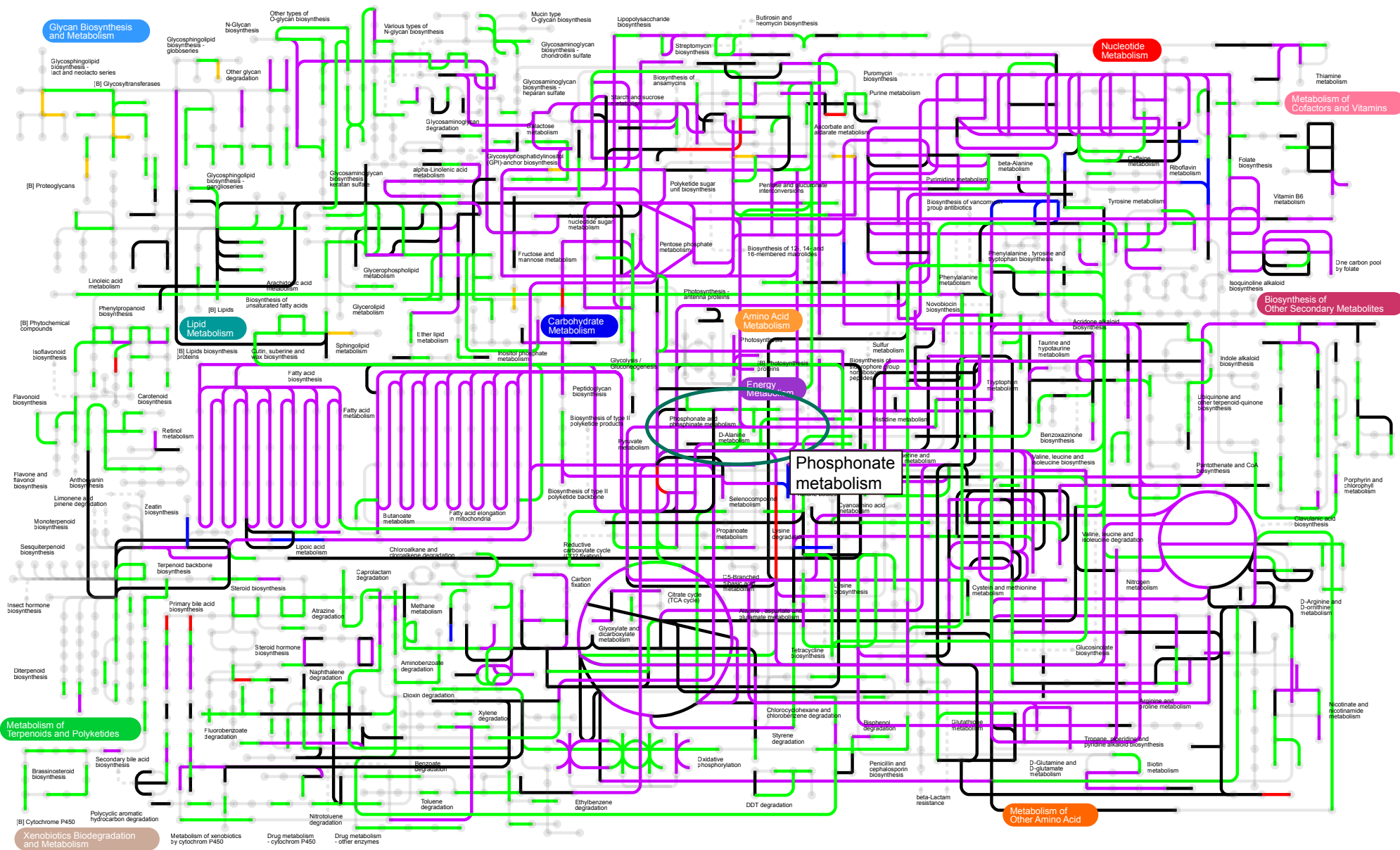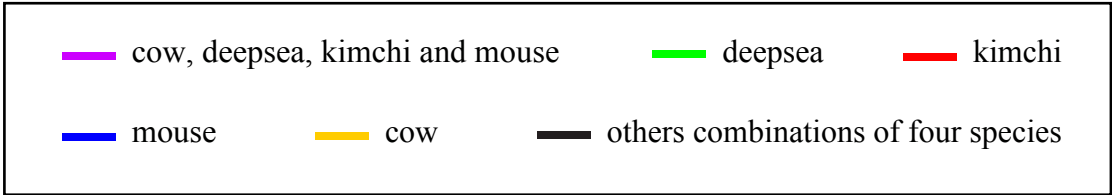

Supplement: Additional file 6: — iPath representation of common and unique enzymes in a global metabolism map from KEGG. The global metabolic map was generated using the online iPath tool [83] with reactions coloured according to their presence in different metatranscriptomic datasets (see inset key). (PDF 1815 kb) [file 40168_2015_146_MOESM6_ESM.pdf]

Cow

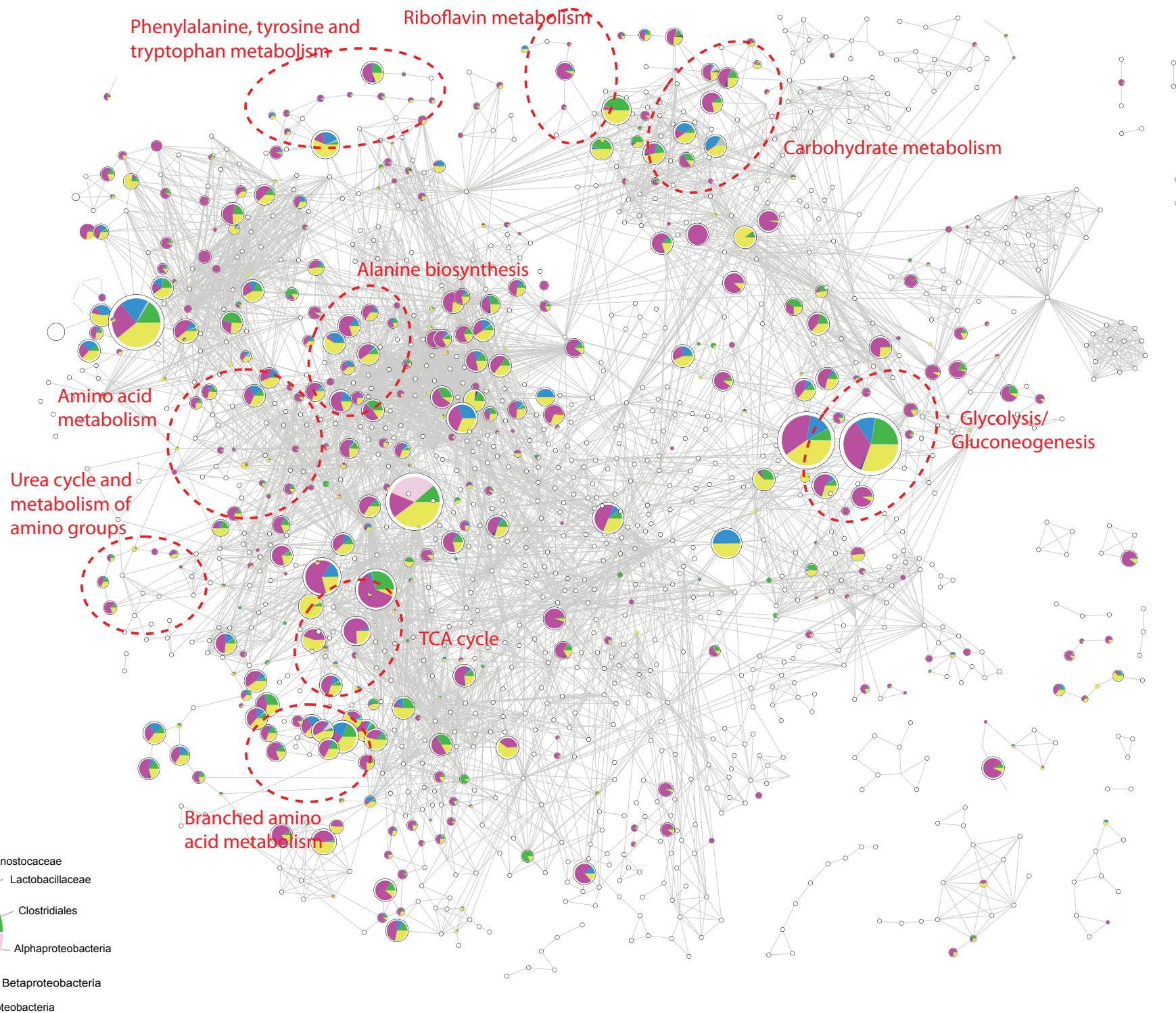

Supplement: Additional file 7: — Global metabolic network indicating taxonomic representation of metabolic activities within the cow rumen dataset. Global metabolic network indicating taxonomic representation of metabolic activities within the cow rumen dataset. Pie charts indicate the relative proportion of each taxon, size of pie chart indicates relative expression (see key). Indicated are specific metabolic pathways. (PDF 1162 kb) [file 40168_2015_146_MOESM7_ESM.pdf]

Kimchi

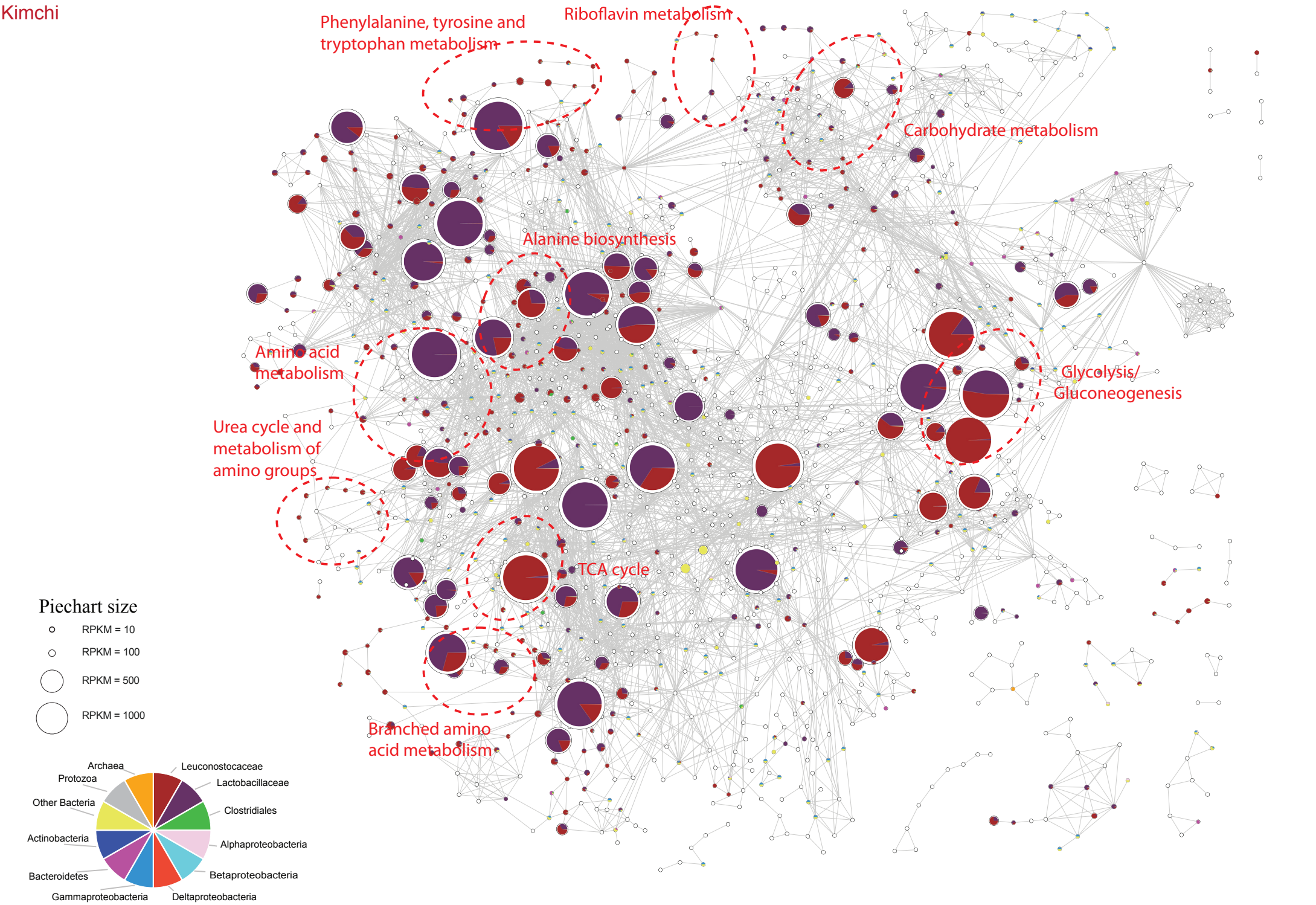

Supplement: Additional file 8: — Global metabolic network indicating taxonomic representation of metabolic activities within the kimchi. Global metabolic network indicating taxonomic representation of metabolic activities within the kimchi dataset. Pie charts indicate the relative proportion of each taxon, size of pie chart indicates relative expression (see key). Indicated are specific metabolic pathways. (PDF 1352 kb) [file 40168_2015_146_MOESM8_ESM.pdf]

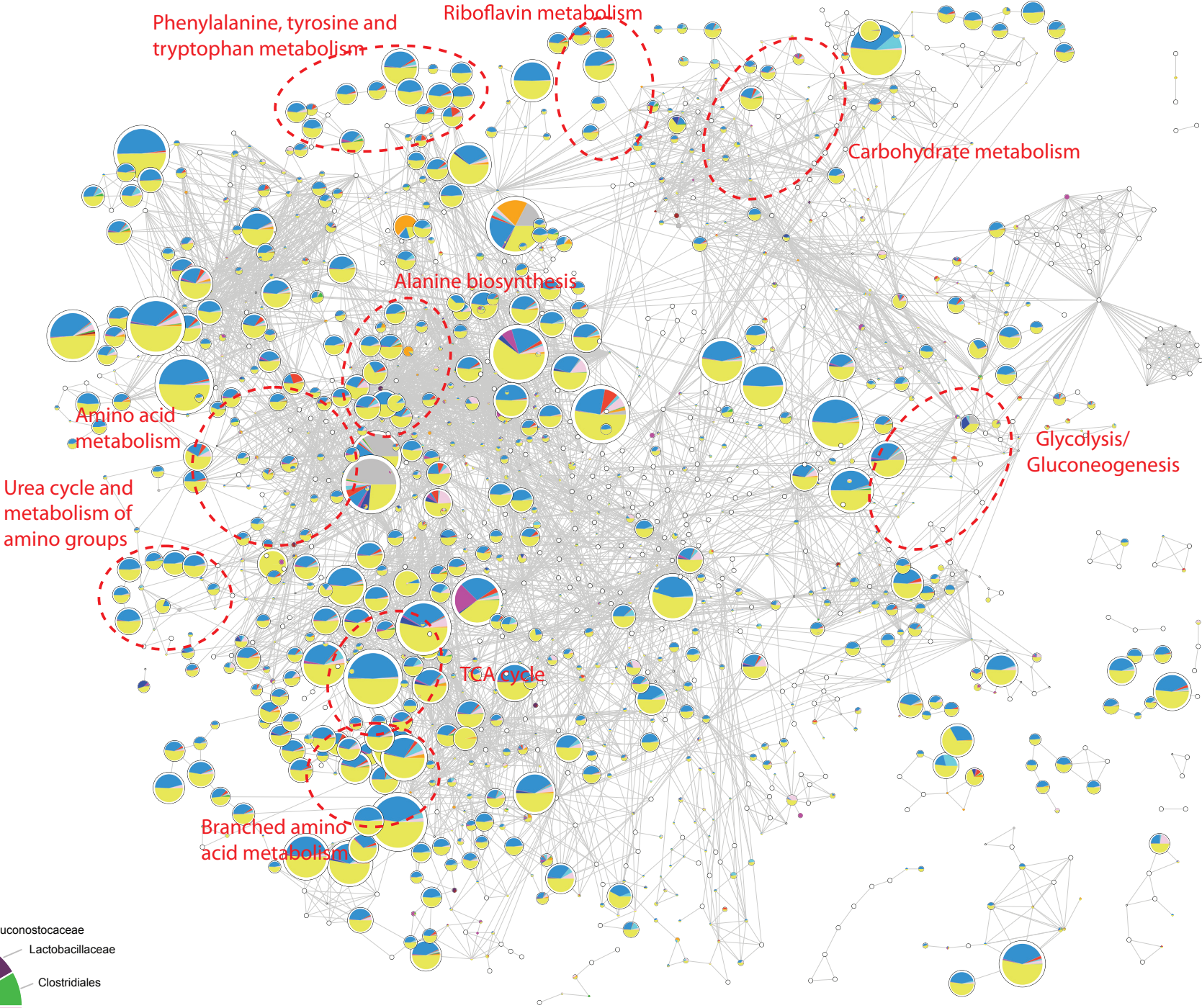

Piechart size

- RPKM = 10
- RPKM = 100
- RPKM = 500
- RPKM = 1000

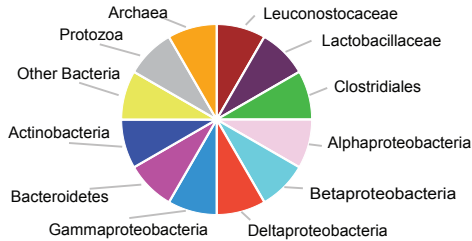

Supplement: Additional file 9: — Global metabolic network indicating taxonomic representation of metabolic activities within the deepsea dataset. Global metabolic network indicating taxonomic representation of metabolic activities within the deepsea dataset. Pie charts indicate the relative proportion of each taxon, size of pie chart indicates relative expression (see key). Indicated are specific metabolic pathways. (PDF 2066 kb) [file 40168_2015_146_MOESM9_ESM.pdf]

Mouse

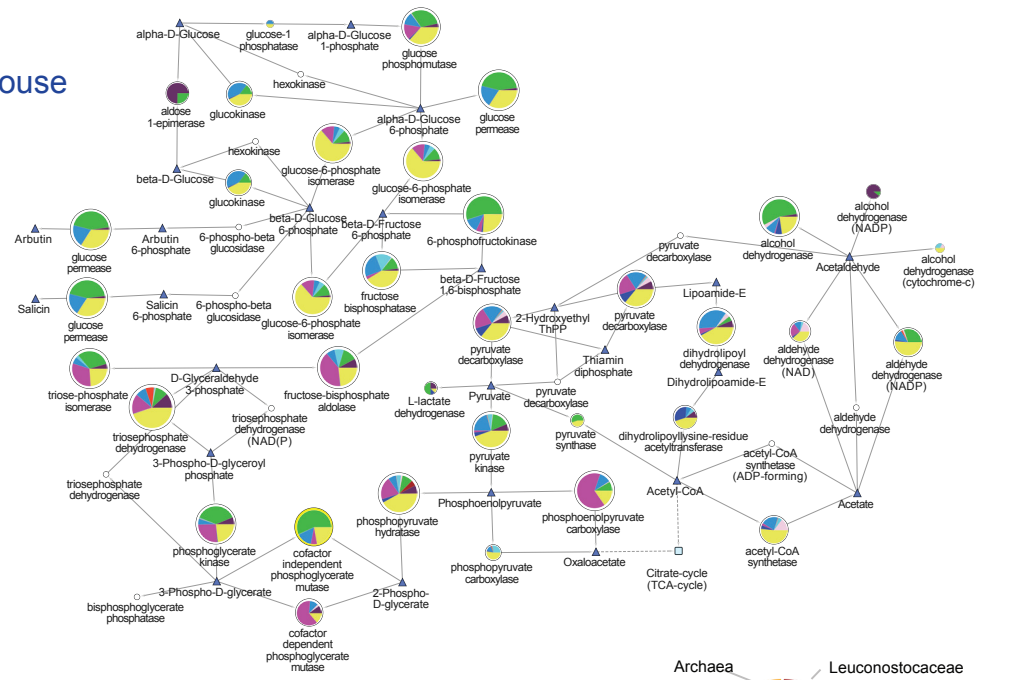

Kimchi

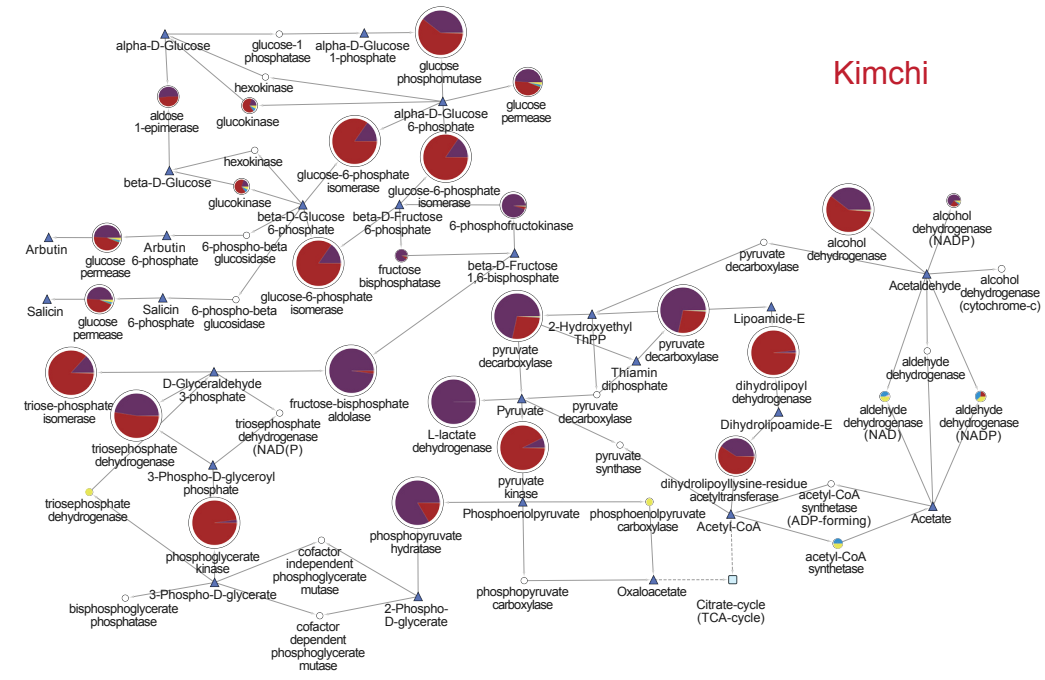

Cow

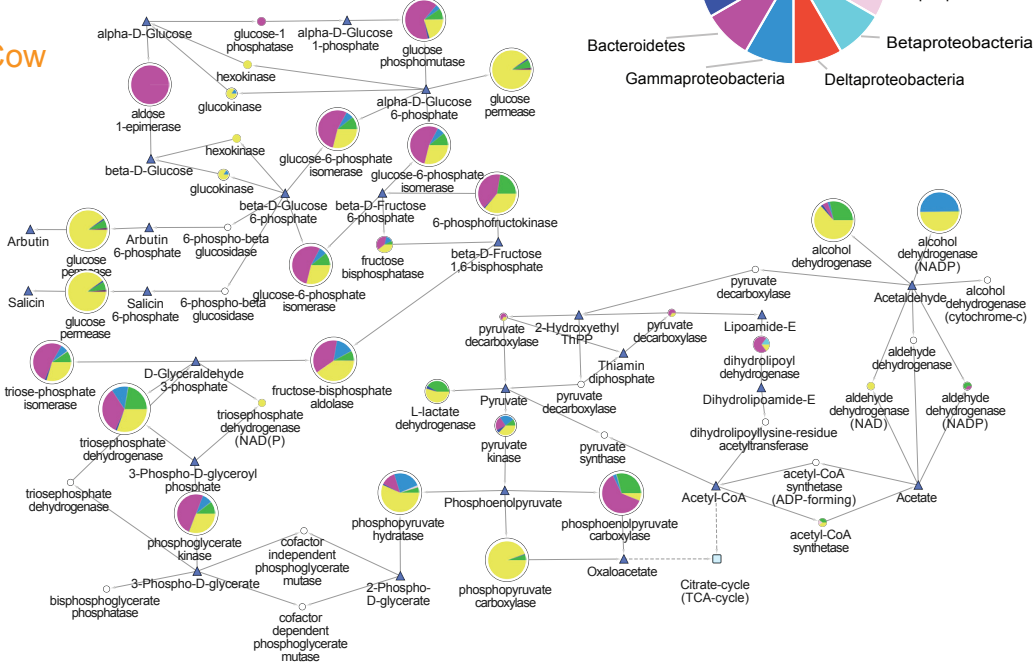

Deepsea

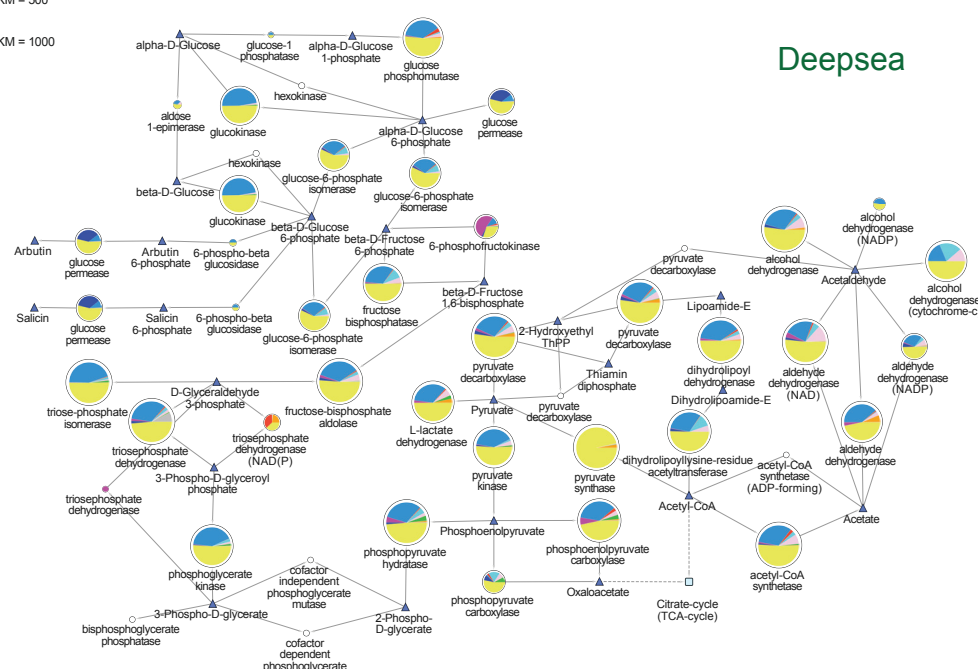

Piechart size

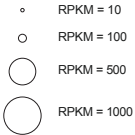

Supplement: Additional file 10: — Detailed views of taxonomic contributions to specific components of the glycolysis/gluconeogenesis pathway for four metatranscriptomic datasets. Each schematic indicates the taxonomic representation of enzymatic activities involved in the glycolysis/gluconeogenesis pathways for four metatranscriptome datasets: mouse, kimchi, cow and deepsea. Pie charts indicate enzymes, with coloured sectors indicating the relative proportion of each taxon, size of pie chart indicates relative expression (see key). Small triangles indicate substrates with links indicating enzyme-substrate relationships. (PDF 635 kb) [file 40168_2015_146_MOESM10_ESM.pdf]

Mouse

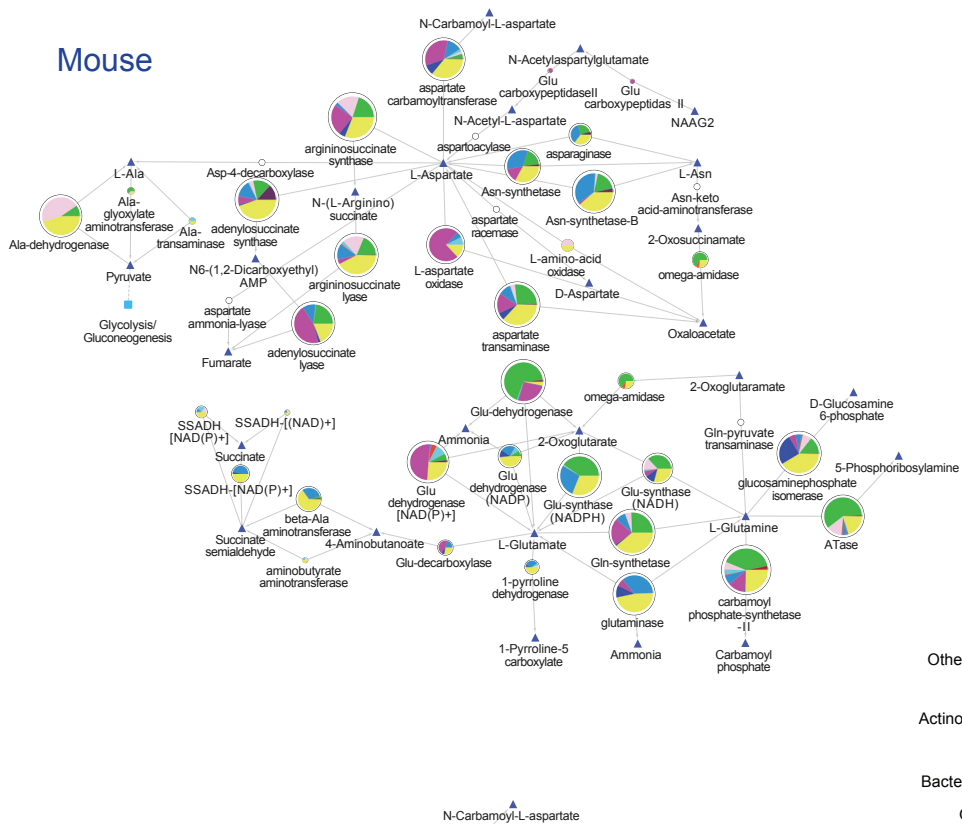

Kimchi

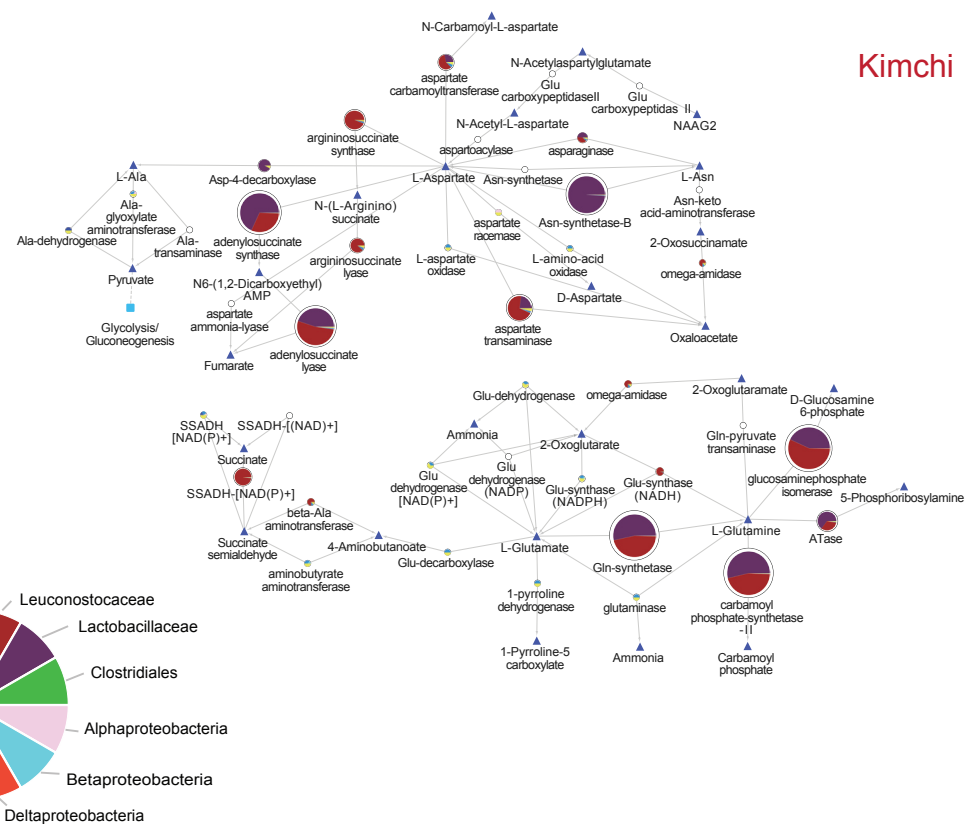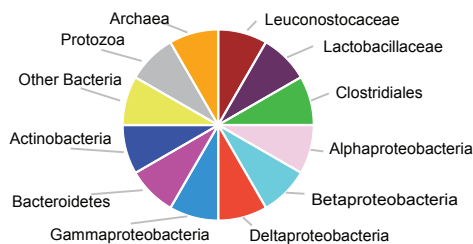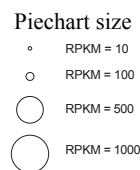

Cow

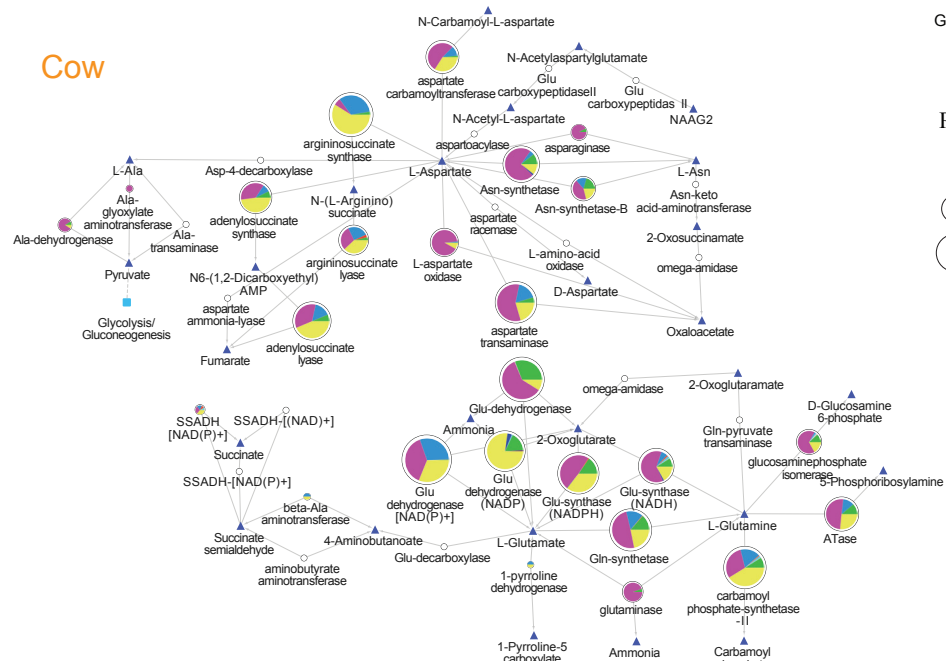

Deepsea

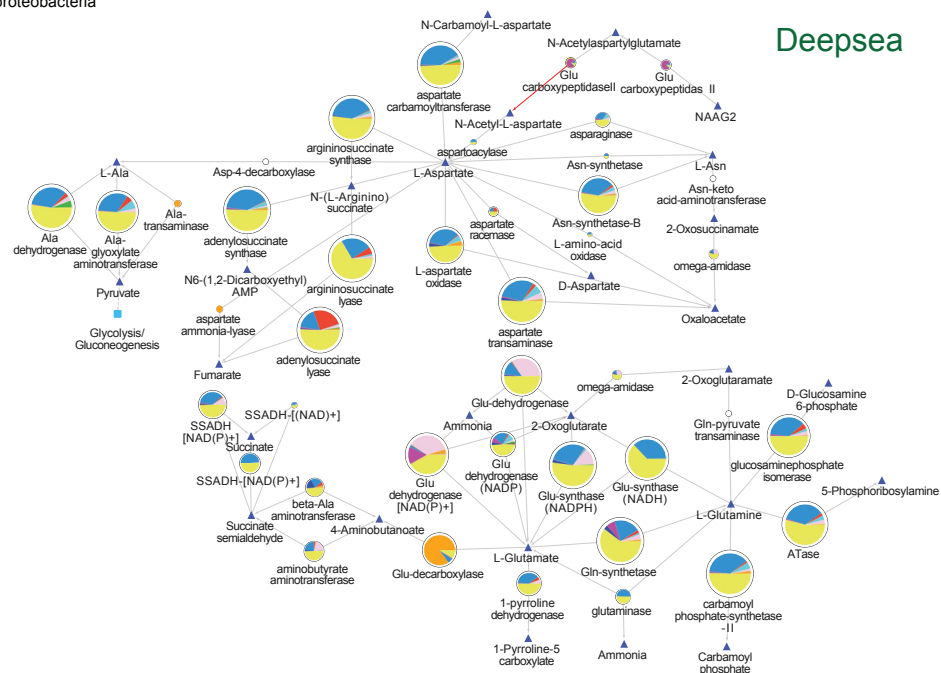

Supplement: Additional file 11: — Detailed views of taxonomic contributions to specific components of the alanine, aspartate and glutamate pathways for four metatranscriptomic datasets. Each schematic indicates the taxonomic representation of enzymatic activities involved in the alanine, aspartate and glutamate pathways for four metatranscriptome datasets: mouse, kimchi, cow and deep sea. Pie charts indicate enzymes, with coloured sectors indicating the relative proportion of each taxon, size of pie chart indicates relative expression (see key). Small triangles indicate substrates with links indicating enzyme-substrate relationships. (PDF 658 kb) [file 40168_2015_146_MOESM11_ESM.pdf]

# Flagella and Chemotaxis

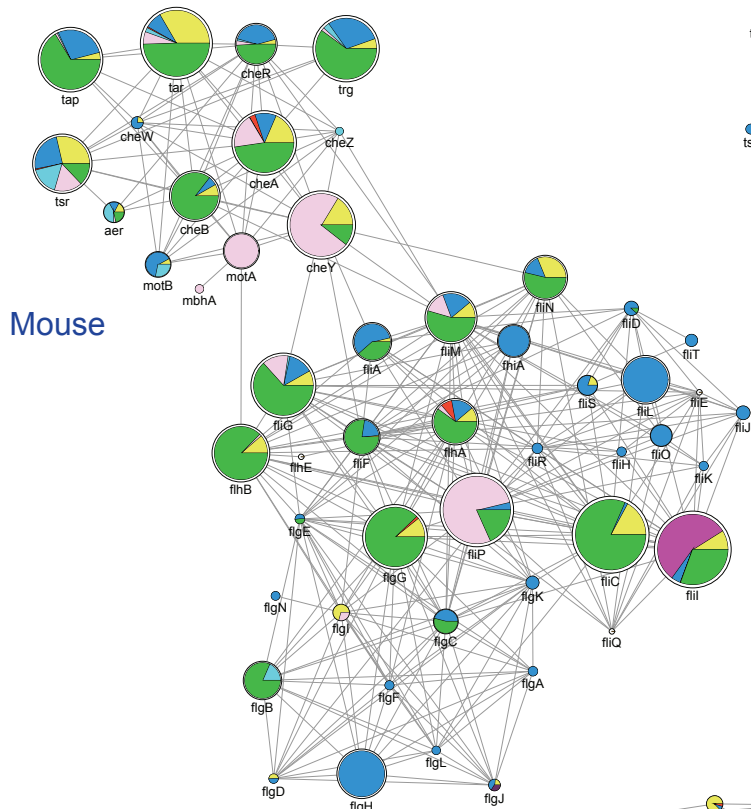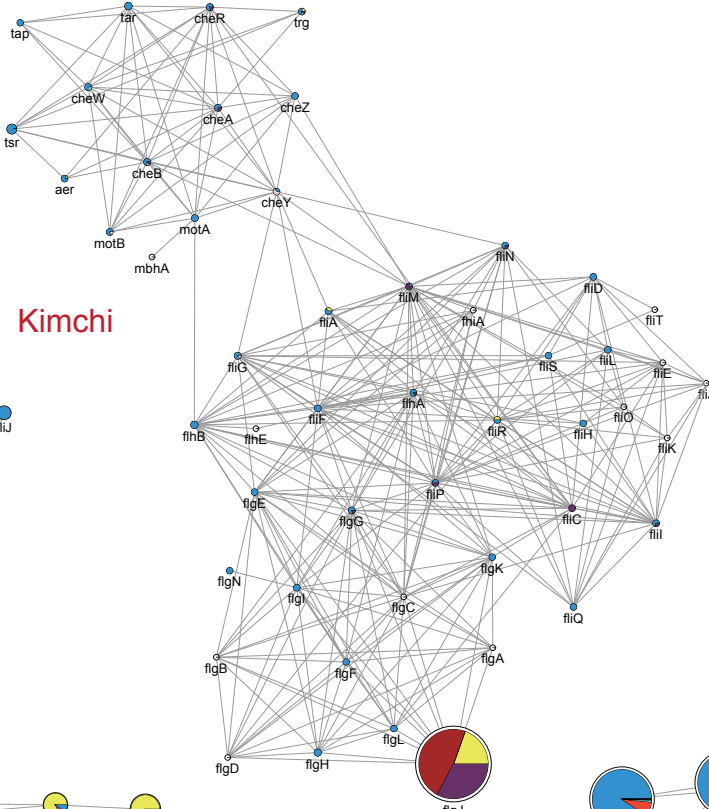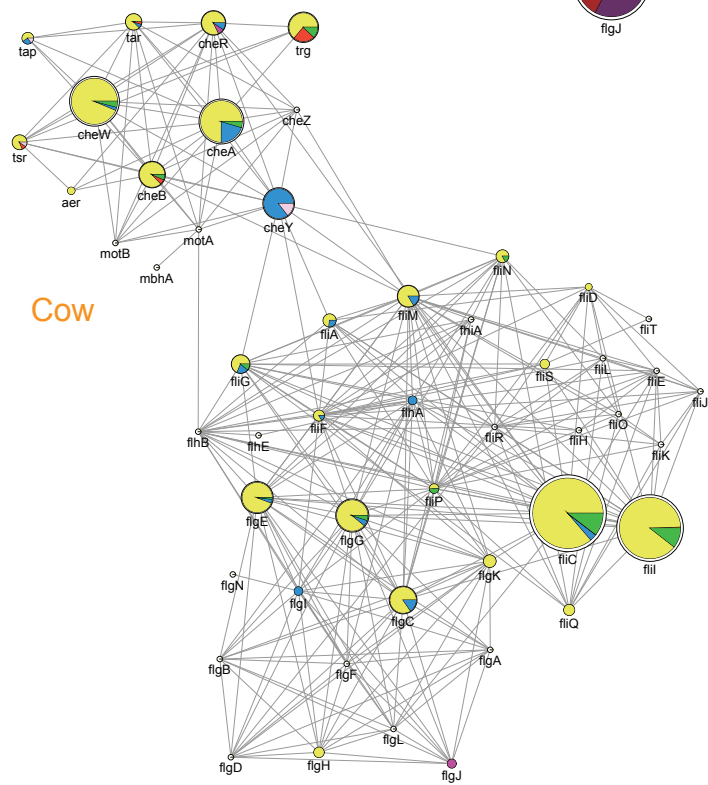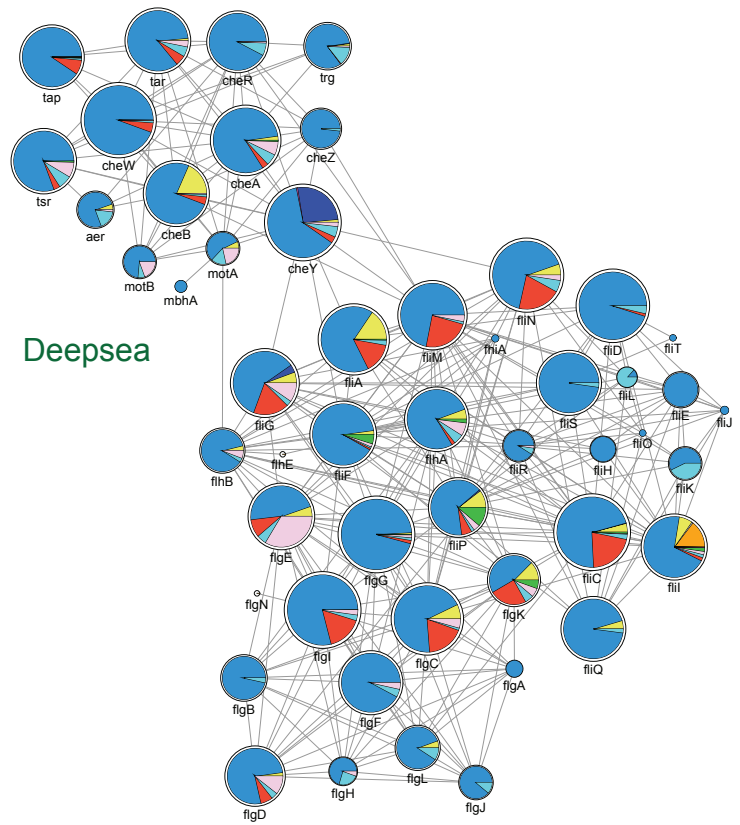

## Piechart size

- RPKM = 10
- RPKM = 100
- RPKM = 500
- RPKM = 1000

## Node Pie Color

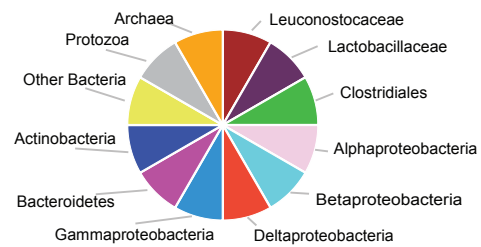

Supplement: Additional file 12: — Taxonomic contributions to flagella biosynthesis and chemotaxis modules as defined through protein-interaction networks for the four well sampled datasets. Each network indicates the taxonomic representation of components of flagella biosynthesis and chemotaxis modules as defined through protein-interaction networks for four metatranscriptome datasets: mouse, kimchi, cow and deepsea. Pie charts indicate genes, with coloured sectors indicating the relative contribution to gene expression for each taxon, size of pie chart indicates relative expression (see key). (PDF 202 kb) [file 40168_2015_146_MOESM12_ESM.pdf]

## Hydrogenases

## Mouse

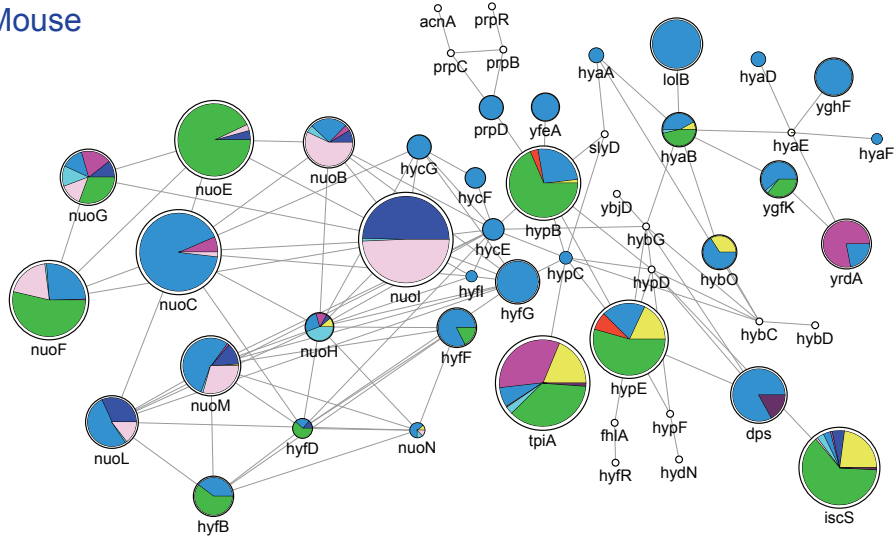

## Cow

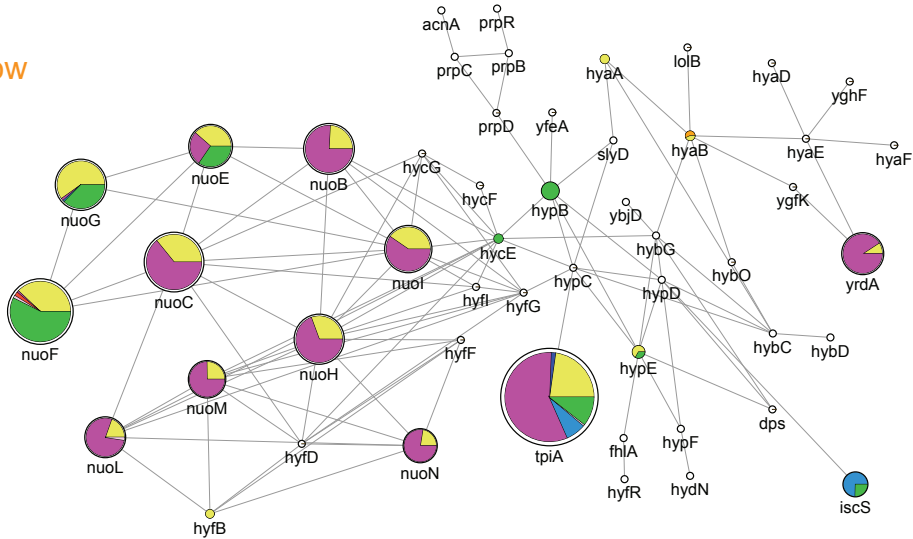

## Kimchi

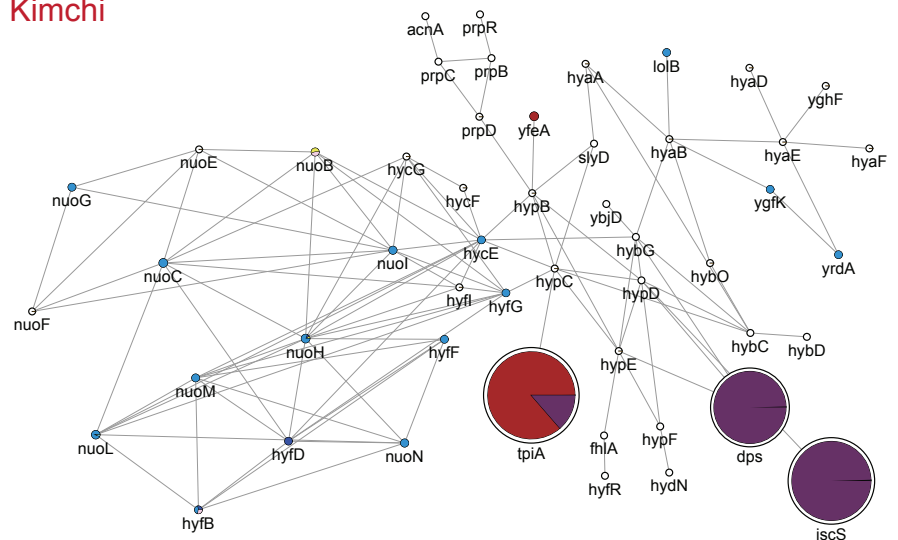

## Deepsea

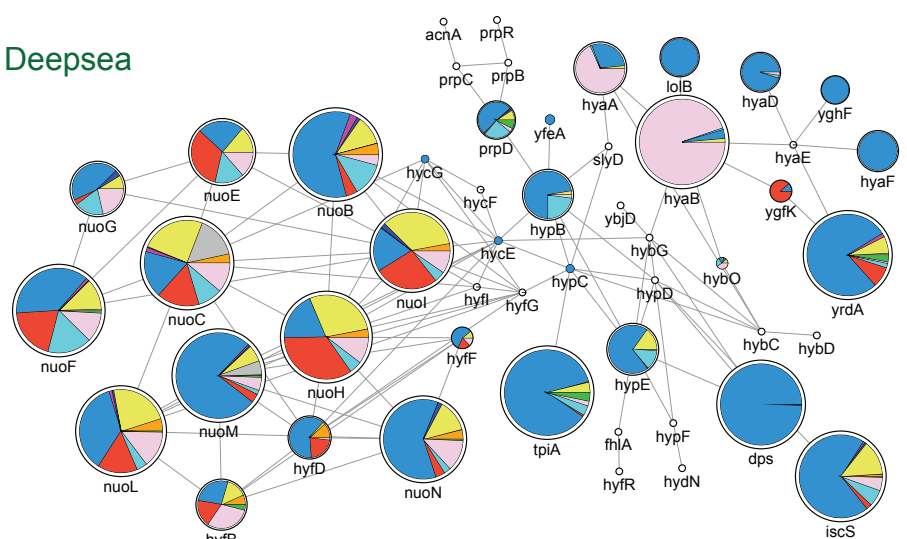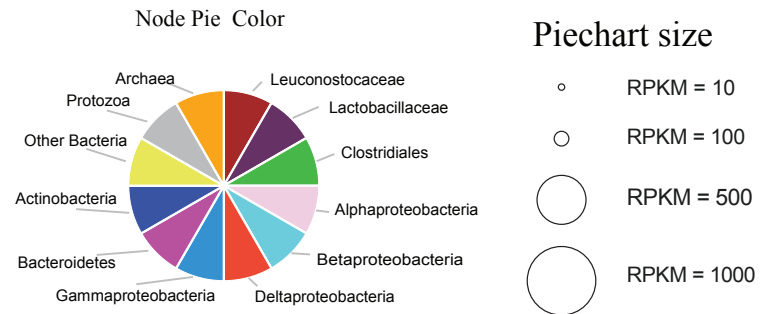

Supplement: Additional file 13: — Taxonomic contributions to select hydrogenase modules as defined through protein-interaction networks for the four well sampled datasets. Each network indicates the taxonomic representation of components of hydrogenases as defined through protein-interaction networks for four metatranscriptome datasets: mouse, kimchi, cow and deep sea. Pie charts indicate genes, with coloured sectors indicating the relative contribution to gene expression for each taxon, size of pie chart indicates relative expression (see key). (PDF 190 kb) [file 40168_2015_146_MOESM13_ESM.pdf]
